# A Bioorthogonal Antidote Against the Photosensitivity after Photodynamic Therapy

**DOI:** 10.1002/advs.202306207

**Published:** 2023-12-31

**Authors:** Evelyn Y. Xue, Caixia Yang, Yimin Zhou, Dennis K. P. Ng

**Affiliations:** ^1^ Department of Chemistry The Chinese University of Hong Kong Shatin, N.T. Hong Kong China

**Keywords:** antidote, bioorthogonal chemistry, boron dipyrromethene, photodynamic therapy, photosensitivity

## Abstract

As an effective and non‐invasive treatment modality for cancer, photodynamic therapy (PDT) has attracted considerable interest. With the recent advances in the photosensitizing agents, the fiber‐optic systems, and other aspects, its application is extended to a wide range of superficial and localized cancers. However, for the few clinically used photosensitizers, most of them suffer from the drawback of causing prolonged photosensitivity after the treatment. As a result, post‐PDT management is also a crucial issue. Herein, a facile bioorthogonal approach is reported that can effectively suppress this common side effect of PDT in nude mice. It involves the use of an antidote that contains a black‐hole quencher BHQ‐3 conjugated with a bicyclo[6.1.0]non‐4‐yne (BCN) moiety and a tetrazine‐substituted boron dipyrromethene‐based photosensitizer. By using tumor‐bearing nude mice as an animal model, it is demonstrated that after PDT with this photosensitizer, the administration of the antidote can effectively quench the photodynamic activity of the residual photosensitizer by bringing the BHQ‐3 quencher close to the photosensitizing unit through a rapid click reaction. It results in substantial reduction in skin damage upon light irradiation. The overall results demonstrate that this simple and facile strategy can provide an effective means for minimizing the photosensitivity after PDT.

## Graphical abstract

Prolonged photosensitivity remains a very common side effect of photodynamic therapy caused by the residual photosensitizer in the body. Herein, a facile and efficient strategy is reported to address this problem, using a bioorthogonal antidote to quench its photodynamic activity after the treatment. The substantial reduction in skin damage is demonstrated in nude mice.

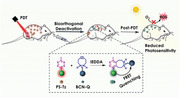

## 1 Introduction

Photodynamic therapy (PDT) has emerged as a promising treatment modality for cancer.^[^ ^1^ ^]^ It involves the light irradiation of a photosensitizer localized in malignant tumor, followed by the interaction with the endogenous oxygen to generate cytotoxic reactive oxygen species (ROS), such as singlet oxygen. Apart from the direct killing of cancer cells through necrosis, apoptosis, and/or other forms of regulated cell death, the ROS generated can also damage the tumor vasculature, indirectly eradicating the tumor by disrupting the oxygen and nutrient supply.^[^ ^2^ ^]^ As the three components are individually non‐toxic, the treatment exhibits minimal invasiveness and less systemic toxicity compared with the traditional anticancer therapies. The photodynamic action could be confined to the target site through precise delivery of the light, endowing a high spatiotemporal selectivity to this modality. By using tailor‐made photosensitizing materials and advanced light technologies, it could also overcome the light penetration barrier.^[^ ^3^ ^]^ Owing to these advantages, PDT has received growing attention as an innovative and effective procedure for cancer treatment, and there have been extensive studies to address the various challenges of PDT in recent years with a view to promoting its clinical applications.^[^ ^4^ ^]^

Since the first PDT drug Photofrin (or porfimer sodium) being approved in Canada in 1993,^[^ ^1c^ ^]^ considerable efforts have been devoted to developing more potent and safer photosensitizers with fewer side effects.^[^ ^5^ ^]^ To date, only a handful of photosensitizers have been approved for oncological use against a range of superficial and localized cancers, most of which are based on porphyrins or chlorins.^[^ ^1d^ ^]^ For most of these clinically used photosensitizing agents, they suffer from the low tumor selectivity and slow metabolism. As a result, they tend to accumulate in the skin after systemic administration, causing the horrendous effect of prolonged photosensitivity.^[^ ^6^ ^]^ Taking Photofrin as an example, the residual drug is present in all parts of the skin, which inevitably causes cutaneous toxicity upon exposure to sunlight.^[^ ^7^ ^]^ According to the guidelines of U.S. Food and Drug Administration, all patients receiving this drug must avoid exposure of skin and eyes to direct sunlight or bright indoor light for at least 30 days (up to 90 days or more for some patients) to allow complete clearance of the drug from the body.^[^ ^8^ ^]^ Hence, the post‐treatment phototoxicity of photosensitizers remains a challenge that limits the clinical use of this modality.

With the goal of circumventing this problem, various strategies have been explored in laboratories. Apart from the approach of using tumor‐targeting ligands to promote the active uptake of photosensitizers by cancer cells,^[^ ^9^ ^]^ a wide range of activatable photosensitizers have also been developed, which ideally can be activated only by the stimuli in the tumor microenvironment, making them non‐toxic at the non‐target sites even upon light irradiation.^[^ ^10^ ^]^ However, the actual outcome of employing these “smart” photosensitizers is affected by their intrinsic quenching efficiency, the extent of activation, the relevance of the stimuli as tumor biomarkers, as well as the specificity of the activation. As the difference in levels of most of the stimuli in cancer cells and normal cells is somewhat subtle, non‐specific activation is usually unavoidable.

Recently, the concept of self‐degradable photosensitizers has been proposed to address the post‐PDT safety problem. Generally, these photosensitizers lose their photoactivities through degradation by the ROS generated or those present intrinsically in the cells (e.g., ClO^–^). A series of these photosensitizers based on the host‐guest complexes of a boron dipyrromethene (BODIPY) and cucurbit[7]uril^[^ ^11^ ^]^ or pillar[5]arene,^[^ ^12^ ^]^ methylene blue analogues,^[^ ^13^ ^]^ anthracene‐bridged donor‐acceptor chromophores,^[^ ^14^ ^]^ or aggregation‐induced emission dyes^[^ ^15^ ^]^ have been reported. As light is generally required for the degradation of these photosensitizers, it is expected that the residual photosensitizers in the body cannot be degraded in the dark and can still cause photosensitivity upon exposure to light despite the effect may be attenuated along with the photodegradation. Therefore, it is of utmost importance to develop effective strategies for deactivation of photosensitizers after the photodynamic treatment.

To this end, Li et al. have recently developed a family of supramolecular organic frameworks^[^ ^16^ ^]^ and naphthalene‐incorporated tetracationic cyclophanes^[^ ^17^ ^]^ that can adsorb some clinically used photosensitizers, including Photofrin, HiPorfin, Talaporfin, and Chlorin e6 in the body of mice through host‐guest interactions. It has been found that the post‐treatment of these hosts can suppress the sunlight‐induced skin phototoxicity without affecting the PDT efficacy. We report herein an alternative strategy for deactivation of the residual photosensitizer through covalent bioorthogonal coupling with an antidote containing an effective quencher. Bioorthogonal chemistry involves chemical reactions that can take place specifically and efficiently under physiological conditions without interfering with the native biochemical processes.^[^ ^18^ ^]^ By using a range of sophisticated click reactions, such as the copper‐catalyzed alkyne–azide cycloaddition (CuAAC), Staudinger ligation, strain‐promoted alkyne‐azide cycloaddition (SPAAC), and inverse electron‐demand Diels‐Alder reaction (IEDDA), biomolecules can be studied in their native environments and manipulated for various applications, including protein and glycan imaging, identification of active enzymes, targeted drug delivery, and high‐throughput screening of molecules against proteins.^[^ ^19^ ^]^ This approach has also been widely used for activation of fluorescent probes, photosensitizers, molecular drugs, and proteins in living systems for theranostic applications.^[^ ^19^, ^20^ ^]^ However, to the best of our knowledge, bioorthogonal chemistry has rarely been reported for deactivation of the residual drugs to reduce their side effects, including for post‐PDT management.^[^ ^21^ ^]^

## 2 Results and Discussion

### 2.1 Working Principle

**Figure 1:**
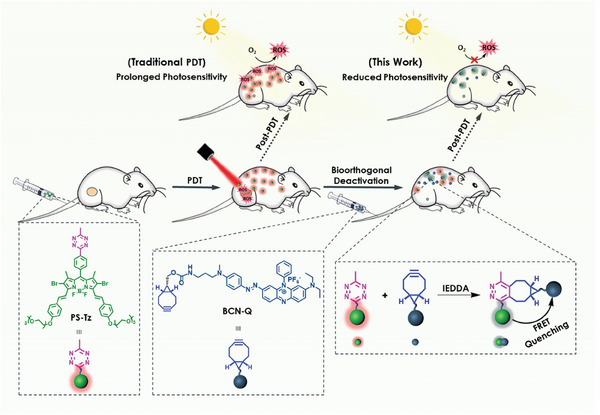
Working principle of the bioorthogonal antidote **BCN‐Q** for deactivation of the residual photosensitizer **PS‐Tz** after the photodynamic treatment.

**Figure** **1** illustrates the concept of this bioorthogonal deactivating approach. It involves the use of a photosensitizer substituted with a tetrazine unit (**PS‐Tz**) and a bicyclo[6.1.0]non‐4‐yne (BCN) conjugated with a quencher (**BCN‐Q**). After PDT, the residual photosensitizer in the body, particularly on the skin, inevitably generates ROS upon exposure to light causing photosensitivity. By injecting the BCN‐modified quencher, it can “click” with the residual tetrazine‐substituted photosensitizer through the highly efficient IEDDA reaction between the two components.^[^ ^22^ ^]^ The proximal quenching unit then effectively inhibits the photodynamic activity of the photosensitizer, thereby preventing the photosensitivity after the treatment. Thus, the BCN‐modified quencher **BCN‐Q** can function as a bioorthogonal antidote against this common side effect of PDT. As the photosensitizing unit is covalently linked to the quencher, the resulting conjugate is expected to be more robust than the non‐covalent counterpart in the biological systems, which might undergo displacement and subsequently reactivation of the photosensitizer.

### 2.2 Molecular Design and Synthesis

Owing to the tunable spectroscopic and photophysical properties, as well as the ease of chemical modification and high stability, BODIPYs are highly versatile photosensitizers for PDT.^[^ ^23^ ^]^ Therefore, a BODIPY dye with an extended conjugation was selected as the photosensitizer for this study, enabling it responsive in the far‐red region. This compound, labeled as **PS‐Tz** (Figure 1), was prepared in 38% yield by Knoevenagel condensation of our previously reported tetrazine‐substituted BODIPY **1** ^[^ ^24^ ^]^ and the corresponding triethylene glycol monomethyl ether‐substituted benzaldehyde **2** ^[^ ^25^ ^]^ (Scheme S1, Supporting Information) and characterized with NMR spectroscopy and electrospray ionization (ESI) mass spectrometry (Figures S1,S2, Supporting Information). The tetrazine unit was introduced to facilitate the coupling with the BCN‐substituted quencher via the IEDDA reaction.^[^ ^22^ ^]^ It is worth mentioning that while a tetrazine moiety can effectively quench the photoactivities of non‐π‐extended BODIPYs,^[^ ^24^, ^26^ ^]^ it is not a good quencher for distyryl BODIPYs.^[^ ^27^ ^]^ It has been reported that tetrazines cannot effectively quench the fluorescence of dyes that emit in the far‐red and near‐infrared region.^[^ ^28^ ^]^

For the antidote **BCN‐Q** (Figure 1), it contains a BCN moiety for bioorthogonal coupling and a black‐hole quencher BHQ‐3 moiety. With a polyaromatic azo skeleton, this non‐emissive azo dye is a well‐known dark quencher for red‐ and far‐red‐emitting fluorophores through the Förster resonance energy transfer (FRET) mechanism owing to the appropriate spectral overlap, involving photochemical isomerization of the azo bridge to provide an effective non‐emissive relaxation pathway.^[^ ^29^ ^]^ Apart from the fluorescence emission, the ROS generation ability of a range of photosensitizers can also be effectively quenched by this dye via FRET.^[^ ^30^ ^]^ This antidote was prepared according to our previously reported procedure.^[^ ^30d^ ^]^ Its stability in phosphate‐buffered saline (PBS) and the cell culture medium Dulbecco's modified Eagle medium (DMEM) supplemented with fetal bovine serum (FBS) (10%) was examined using high‐performance liquid chromatography (HPLC). In both media, the chromatogram was virtually unchanged over a period of 14 days (Figure S3, Supporting Information), indicating that this conjugate possesses high stability in these biological media.

### 2.3 Bioorthogonal Quenching in Solution

**Figure 2:**
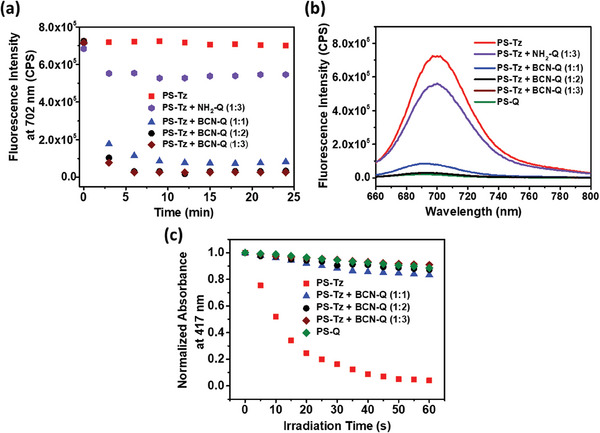
a) Change in the fluorescence intensity at 702 nm of **PS‐Tz** (1 µm) with or without the presence of different concentrations of **BCN‐Q** (1, 2, and 3 µm) or **NH_2_‐Q** (3 µm) over a period of 24 min (λ_ex_ = 610 nm). b) Comparison of the fluorescence spectra of **PS‐Q** (1 µm) and the above mixtures recorded at 6 min after the mixing (λ_ex_ = 610 nm). c) Comparison of the rates of decay of DPBF (initial concentration = 30 µm) sensitized by **PS‐Q** (1 µm), **PS‐Tz** (1 µm), and mixtures of **PS‐Tz** (1 µm) and **BCN‐Q** (1, 2, and 3 µm) after mixing for 6 min. The mixtures were irradiated (λ > 610 nm) during the measurements. The solvent was PBS in the presence of 0.1% Tween 80 (v/v) for all the above measurements.

The bioorthogonal quenching of **PS‐Tz** by **BCN‐Q** was first examined by monitoring the change in fluorescence spectrum of **PS‐Tz** (1 µm) in PBS in the presence of 0.1% Tween 80 (v/v) with or without the presence of different concentrations of **BCN‐Q** (1, 2, and 3 µm) over a period of 24 min. The surfactant Tween 80 was added to increase the solubility and reduce the aggregation of the compounds in this aqueous medium. It was found that in the absence of **BCN‐Q**, the spectrum was virtually unchanged over this period of time (Figure S4a, Supporting Information). Upon addition of **BCN‐Q** (1, 2, and 3 µm), the fluorescence band at ca. 700 nm was diminished largely and almost spontaneously (Figure S4b–d, Supporting Information). As shown in **Figure** **2**a, which depicts the time‐dependent variation of the fluorescence intensity at 702 nm, when 1 µm of **BCN‐Q** was added, the intensity was reduced to the minimum after ca. 12 min, while the time was shortened to ca. 6 min when 2 or 3 µm of **BCN‐Q** was used. The effective quenching could be attributed to the covalent linkage to the BHQ‐3 moiety via an IEDDA reaction. To provide further evidence, the commercially available non‐BCN‐substituted BHQ‐3 amine **NH_2_‐Q** and a non‐BHQ‐3‐conjugated BCN derivative **3** ^[^ ^31^ ^]^ were also used for comparison. It was found that in the presence of 3 µm of **NH_2_‐Q**, the fluorescence intensity of **PS‐Tz** (1 µm) was just slightly reduced over the whole period of time (Figure S4e, Supporting Information, and Figure 2a), showing that the click process was essential for effective quenching of the fluorescence of **PS‐Tz**. As expected, without the quenching component, BCN **3** could not significantly change the fluorescence intensity of **PS‐Tz** (Figure S4f, Supporting Information).

To demonstrate the generality of this approach and for comparison, we also used our previously reported azide‐modified distyryl BODIPY,^[^ ^32^ ^]^ labeled as **PS‐N_3_**, instead of **PS‐Tz** as the photosensitizer. With a reactive azide group, this compound was expected to couple with **BCN‐Q** efficiently via the SPAAC reaction.^[^ ^33^ ^]^ Therefore, it could also be used to demonstrate the bioorthogonal deactivation. Similarly, we monitored the change in fluorescence spectrum of this compound (1 µm) with or without the presence of **BCN‐Q** (1, 2, and 3 µm) over a period of 24 min. As expected, its fluorescence at ca. 720 nm was also largely reduced in the presence of **BCN‐Q**, and the rate of decrease was slightly faster when a higher concentration of **BCN‐Q** was used (Figure S5, Supporting Information). As shown in Figure S5e (Supporting Information), more than 15 min was required to attain the lowest intensity, showing that the rate of quenching for **PS‐N_3_** was slightly slower than that for **PS‐Tz** (Figure 2a), which could be attributed to the less efficient SPAAC reactions compared with the IEDDA reactions.^[^ ^34^ ^]^ Owing to the more efficient quenching by **BCN‐Q**, **PS‐Tz** was used for all the subsequent studies.

As the quenching of the fluorescence could be attributed to the formation of the clicked product, the conjugate of **PS‐Tz** and **BCN‐Q** was prepared for comparison. As shown in Scheme S2 (Supporting Information), the clicked product labeled as **PS‐Q** could be prepared readily (in 95% yield) by mixing the two components in CH_3_CN, followed by purification by reserve‐phase HPLC (Figure S6, Supporting Information) and characterization by ESI mass spectrometry (Figure S7, Supporting Information). As expected, the fluorescence of this conjugate in the aforementioned aqueous medium was virtually vanished as shown in Figure 2b, which also includes the fluorescence spectra of **PS‐Tz** (1 µm) with or without the presence of different concentrations of **BCN‐Q** (1, 2, and 3 µm) or **NH_2_‐Q** (3 µm) recorded at 6 min after the mixing when the coupling was essentially completed. It can be seen that when 2 or 3 µm of **BCN‐Q** was used, the resulting spectra did not show noticeable fluorescence as in the case of the conjugate **PS‐Q**. Hence, it can be concluded that 2 equiv. of **BCN‐Q** is already sufficient to fully quench the fluorescence of **PS‐Tz** within 6 min, and this amount was used in the biological studies.

To quantitatively determine the quenching efficiency, the fluorescence quantum yields (Φ_f_) of **PS‐Tz** and **PS‐Q** were measured and compared both in *N*,*N*‐dimethylformamide (DMF) and in water with 0.1% Tween 80 (v/v). As shown in Figure S8a (Supporting Information), while a strong fluorescence band was observed at 707 nm for **PS‐Tz**, the fluorescence of **PS‐Q** was negligible as in the case of the quenching component **BCN‐Q** in DMF. The respective fluorescence quantum yields were determined to be 0.36 and 0.001 with reference to zinc(II) phthalocyanine (ZnPc) (Φ_f_ = 0.28).^[^ ^30d^ ^]^ The fluorescence quantum yield of **PS‐Tz** in DMF was very close to those of our previously reported non‐tetrazine‐substituted dibromo distyryl BODIPYs,^[^ ^35^ ^]^ which further supported that the tetrazine unit does not exert a significant quenching effect on the BODIPY core. The results in the aqueous medium were very similar with the value of Φ_f_ decreasing from 0.31 for **PS‐Tz** to 0.009 for **PS‐Q** (**Table** **1**).

**Table 1:** Fluorescence and singlet oxygen generation properties of **PS‐Tz** and **PS‐Q** in DMF and in water with 0.1% Tween 80 (v/v).

Compound	Solvent	λ_em_ (nm)^a)^	Φ_f_ ^b)^	Φ_Δ_
**PS‐Tz**	DMF	707	0.36	0.12^c)^
**PS‐Tz**	water^d)^	702	0.31	0.38^e)^
**PS‐Q**	DMF	—^f)^	0.001	0.002^c)^
**PS‐Q**	water^d)^	697	0.009	0.02^e)^

^a)^ Excited at 610 nm;

^b)^ Relative to ZnPc (Φ_F_ = 0.28 in DMF);

^c)^ Relative to ZnPc (Φ_Δ_ = 0.56 in DMF) using DPBF as the singlet oxygen scavenger;

^d)^ In the presence of 0.1% Tween 80 (v/v);

^e)^ Relative to MB (Φ_Δ_ = 0.52 in water) using ABDA as the singlet oxygen scavenger;

^f)^ The value was too weak to be measured.

Apart from the study of fluorescence quenching, we also investigated the effect of singlet oxygen formation upon the bioorthogonal coupling. As photosensitivity is mainly caused by ROS, the deactivation of photosensitizers in this aspect is of vital importance. Using 1,3‐diphenylisobenzofuran (DPBF) as the singlet oxygen probe,^[^ ^36^ ^]^ the singlet oxygen generation efficiency of **PS‐Tz** (1 µm) was monitored before and after the treatment with different concentrations of **BCN‐Q** (1, 2, and 3 µm) in the same aqueous medium for 6 min. As shown in Figure 2c, the absorbance of the DPBF's absorption at 417 nm decreased rapidly upon light irradiation (λ > 610 nm) for **PS‐Tz**, indicating that it is an efficient photosensitizer. However, after the treatment with **BCN‐Q** for 6 min, the singlet oxygen generation ability of **PS‐Tz** was largely suppressed. The efficiency was comparable with that of the covalent conjugate **PS‐Q**, particularly when 2 or 3 equiv. of **BCN‐Q** was used.

Similarly, the quenching efficiency in this aspect was quantitatively evaluated by comparing the singlet oxygen quantum yields (Φ_Δ_) of **PS‐Tz** and **PS‐Q** in DMF and in water with 0.1% Tween 80 (v/v). For the measurements in DMF, DPBF was again used as the singlet oxygen scavenger^[^ ^36^ ^]^ and ZnPc was used as the reference (Φ_Δ_ = 0.56),^[^ ^30d^ ^]^ while in the aqueous medium, 9,10‐anthrancenediyl‐bis(methylene)dimalonic acid (ABDA) was used as the probe^[^ ^36^ ^]^ with reference to methylene blue (MB) (Φ_Δ_ = 0.52 in water).^[^ ^37^ ^]^ As shown in Figure S9 (Supporting Information), **PS‐Tz** could effectively consume both singlet oxygen scavengers upon irradiation, though its efficiency was lower than that of the reference compounds, from which the Φ_Δ_ values were determined to be 0.12 in DMF and 0.38 in the aqueous medium. In contrast, the singlet oxygen generation efficiency of **PS‐Q** was negligible. The Φ_Δ_ values were decreased to 0.002 and 0.02 in these two media, respectively (Table 1).

As mentioned above, it is believed that the quenching of these photoactivities is mainly through the FRET mechanism. It is based on the substantial spectral overlap between the fluorescence spectrum of **PS‐Tz** and the electronic absorption spectrum of **BCN‐Q** (Figure S8b, Supporting Information) as well as the highly efficient IEDDA reaction that connects the two components almost spontaneously and brings them in close proximity. The latter is expected to be a more important factor governing the quenching efficiency by FRET as reported previously.^[^ ^38^ ^]^ To reveal the possibility of quenching by ground‐state complex formation, the electronic absorption spectra of **PS‐Tz**, **BCN‐Q**, and **PS‐Q** in DMF were compared (Figure S8c, Supporting Information). It was found that the spectrum of **PS‐Q** is essentially a superposition of the sum of the spectra of **PS‐Tz** and **BCN‐Q**. The negligible shift in the absorption maxima suggests that quenching by the ground‐state complex of the photosensitizing unit and the quencher is minimal.^[^ ^30a^ ^]^

### 2.4 In Vitro Bioorthogonal Deactivation

**Figure 3:**
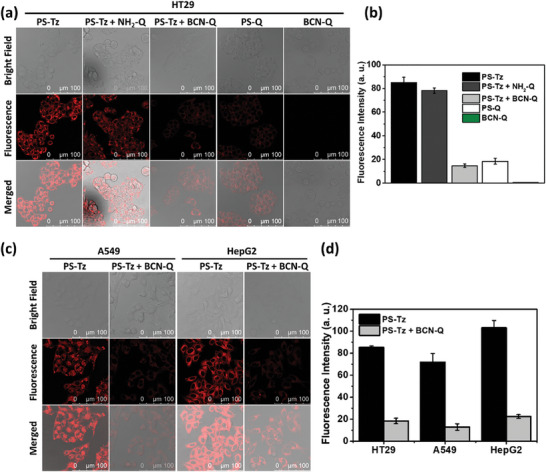
a) Bright field, fluorescence, and the merged confocal images of HT29 cells after incubation with **PS‐Tz** (1 µm) for 6 h and then in the culture medium with or without **NH_2_‐Q** or **BCN‐Q** (2 µm) for 1 h or incubation simply with **PS‐Q** (1 µm) for 6 h or **BCN‐Q** (2 µm) for 1 h. b) Quantified fluorescence intensities for the cells being treated as described in (a). c) Bright field, fluorescence, and the merged confocal images of A549 and HepG2 cells after incubation with **PS‐Tz** (1 µm) for 6 h and then in the culture medium with or without **BCN‐Q** (2 µm) for 1 h. d) Quantified fluorescence intensities for the cells being treated as described in (c). For (b) and (d), data are reported as the mean ± standard error of the mean (SEM) of three independent experiments (*n* = 25 cells).

The bioorthogonal deactivation of **PS‐Tz** by **BCN‐Q** was further demonstrated at the cellular level. HT29 human colorectal adenocarcinoma cells were first incubated with **PS‐Tz** (1 µm) for 6 h and then in the culture medium with or without **NH_2_‐Q** or **BCN‐Q** (2 µm) for 1 h. For comparison, the cells were also incubated with **PS‐Q** (1 µm) for 6 h without the post‐treatment or simply with **BCN‐Q** (2 µm) for 1 h. The corresponding confocal images and quantified intracellular fluorescence intensities are shown in **Figure** **3**a,b, respectively. Bright fluorescence was observed for the cells being treated with **PS‐Tz**, and the fluorescence intensity remained very strong when the cells were post‐treated with **NH_2_‐Q**. In contrast, for the cells being post‐treated with **BCN‐Q**, the fluorescence intensity was reduced by ca. 5‐fold, which was comparable with that for the cells being incubated with **PS‐Q**, indicating that **PS‐Tz** was essentially fully deactivated by **BCN‐Q** inside the cells. As expected, the intracellular fluorescence of the dark quencher **BCN‐Q** was negligible.

A similar study was performed using two other cell lines, namely A549 human lung carcinoma cells and HepG2 human hepatocarcinoma cells. As shown in Figure 3c,d, the intracellular fluorescence intensity of **PS‐Tz** was also largely reduced when the cells were post‐treated with **BCN‐Q**, and the extent (ca. 5‐fold) was similar to that for HT29 cells. The results showed that **BCN‐Q** could effectively deactivate the fluorescence emission of **PS‐Tz** inside a range of cancer cells through bioorthogonal conjugation.

**Figure 4:**
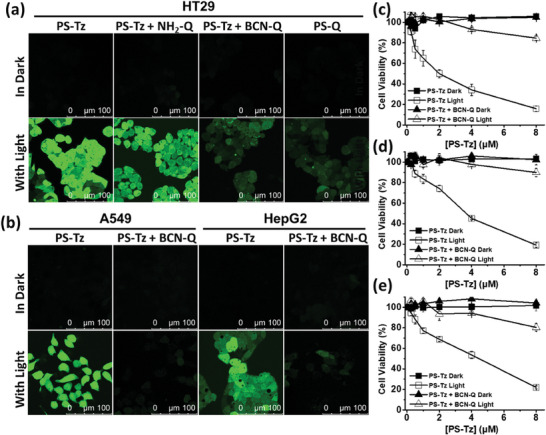
a) Intracellular ROS generation efficiency of **PS‐Tz** (1 µm) with or without post‐treatment with **NH_2_‐Q** or **BCN‐Q** (2 µm) for 1 h and of the conjugate **PS‐Q** (1 µm), as reflected by the fluorescence intensity of DCF, in HT29 cells both in the absence and presence of light irradiation (λ > 610 nm, 23 mW cm^−2^) for 20 min. b) Intracellular ROS generation efficiency of **PS‐Tz** (1 µm) with or without post‐treatment with **BCN‐Q** (2 µm) for 1 h in A549 and HepG2 cells both in the absence and presence of light irradiation (λ > 610 nm, 23 mW cm^−2^) for 20 min. Cytotoxicity of **PS‐Tz** against c) HT29, d) A549, and e) HepG2 cells with or without post‐treatment with **BCN‐Q** (2 equiv.) for 1 h both in the absence and presence of light irradiation (λ > 610 nm, 23 mW cm^−2^, 28 J cm^−2^). Data are reported as the mean ± SEM of three independent experiments, each performed in quadruplicate.

The study was then extended to examine the bioorthogonal deactivation in ROS formation inside the cells, using 2′,7′‐dichlorodihydrofluorescein diacetate (H_2_DCFDA) as the ROS probe.^[^ ^39^ ^]^ Upon internalization, this probe would be deacetylated by the intracellular esterase and subsequently oxidized by the intracellular ROS to form the highly fluorescent 2′,7′‐dichlorofluorescein (DCF), which can be detected readily by confocal microscopy. In this study, the cells were incubated with **PS‐Tz** (1 µm) for 6 h and then in the culture medium with or without **NH_2_‐Q** or **BCN‐Q** (2 µm) for 1 h or incubated simply with **PS‐Q** (1 µm) for 6 h, followed by the incubation with H_2_DCFDA (50 µm) for 30 min. The cells were then either left in the dark or irradiated with red light (λ > 610 nm, 23 mW cm^−2^) for 20 min. **Figure** **4**a shows the confocal fluorescence images of the cells under all these conditions. It can be seen that in the absence of light irradiation, the fluorescence was negligible for all the cells. Upon light irradiation, bright green fluorescence of DCF could be observed for the cells being incubated with **PS‐Tz** and those with post‐treatment with **NH_2_‐Q**. For the cells with post‐treatment with **BCN‐Q**, the fluorescence of DCF was significantly reduced, and the intensity was comparable with that by using the conjugate **PS‐Q** for incubation. The attenuation effect was also observed for A549 and HepG2 cells (Figure 4b), showing that **BCN‐Q** could also effectively deactivate the ROS generation ability of **PS‐Tz** inside the cells.

Since **PS‐Tz** could effectively generate ROS inside the cells upon light irradiation, it was expected to exhibit high photocytotoxicity. To examine this property, all the three cell lines were incubated with different concentrations (up to 8 µm) of **PS‐Tz** for 6 h, followed by the dark or light (λ > 610 nm, 23 mW cm^−2^) treatment for 20 min. As shown in Figure 4c–e, while the photosensitizer was noncytotoxic in the dark, it exhibited high photocytotoxicity. The half‐maximal inhibitory concentration (IC_50_ value) was determined to be 2.0, 3.6, and 4.4 µm for HT29, A549, and HepG2 cells, respectively. Upon post‐incubation of the cells with 2 equiv. of **BCN‐Q** for 1 h, the cells remained viable in the absence of light. Even upon light irradiation, the cell viability was only decreased by at most 20% at a drug dose of 8 µm, showing that the photocytotoxicity of **PS‐Tz** was greatly reduced upon post‐treatment with **BCN‐Q**. These results were consistent with those of the study of intracellular ROS generation as reported above and further demonstrated that the photodynamic activity of **PS‐Tz** could be effectively inhibited by this bioorthogonal antidote.

To reveal the toxicity of the resulting conjugate, the dark and photocytotoxicity of **PS‐Q** was examined against all the three cell lines. As shown in Figure S10a–c (Supporting Information), its cytotoxicity was negligible under all the conditions. For the antidote **BCN‐Q**, its cytotoxicity was also not noticeable (Figure S10d, Supporting Information). These results showed that both the clicked product and the antidote were not cytotoxic. To provide further evidence, hemolysis assay was also performed for the photosensitizer **PS‐Tz**, the antidote **BCN‐Q**, and the conjugate **PS‐Q** using rabbit red blood cells. The cells were incubated with different concentrations of these compounds at 37 °C for 6 h, and then the amount of hemoglobin released was determined spectroscopically. It was found that even at a dose of 40 µm, cell lysis was not noticeable for all these compounds (Figure S11, Supporting Information), showing that they exhibited high blood compatibility.

### 2.5 In Vivo Bioorthogonal Deactivation

**Figure 5:**
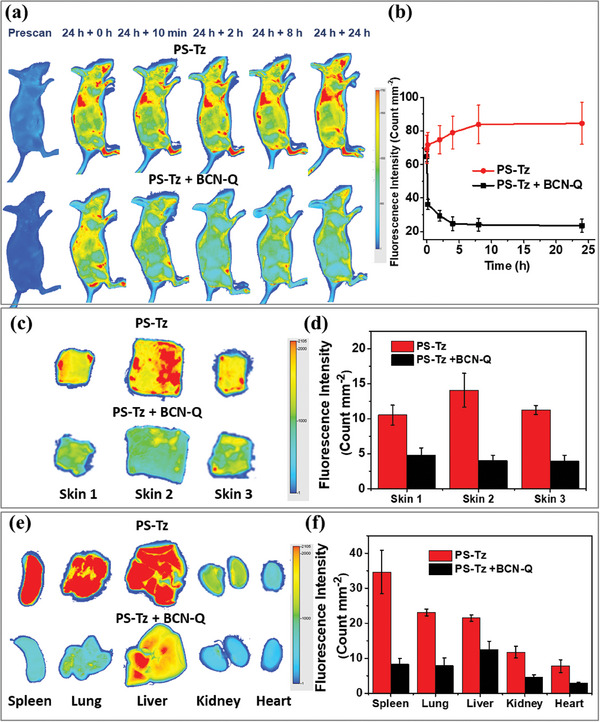
a) Near‐infrared (λ ≥ 700 nm) fluorescence images of the nude mice at 24 h post‐injection of **PS‐Tz** in PBS in the presence of 0.1% Tween 80 (v/v) (20 nmol, 100 µL) and after intravenous injection of **BCN‐Q** in PBS (40 nmol, 200 µL) or simply PBS (200 µL) over a period of further 24 h. b) Change in fluorescence intensity per unit area of the skin at the backside of the mice after the above treatments over a period of 24 h. Ex vivo images and quantified fluorescence intensities of (c,d) the skin tissues and (e,f) some major organs harvested from the above two groups of mice at 24 h post‐injection of **BCN‐Q** or PBS. Skin 1, 2, and 3 are the skin tissues taken from the leg, back, and belly, respectively. Data are reported as the mean ± standard deviation (SD) of three mice in each group.

With these encouraging in vitro results, we further examined the bioorthogonal deactivation effect using a mouse model. Female BALB/c mice were first intravenously injected with **PS‐Tz** in PBS in the presence of 0.1% Tween 80 (v/v) (20 nmol, 100 µL). After 24 h, the mice were administrated intravenously with **BCN‐Q** in PBS (40 nmol, 200 µL) or simply PBS (200 µL) as control. **Figure** **5**a shows the near‐infrared fluorescence images of the mice recorded with an infrared imaging system (excitation wavelength = 680 nm, emission wavelength ≥ 700 nm) at 24 h post‐injection of **PS‐Tz** and after the intravenous injection of **BCN‐Q** or PBS over a period of further 24 h. Strong fluorescence was clearly observed throughout the whole body of the mice at 24 h post‐injection of **PS‐Tz**. After further injection with **BCN‐Q**, the fluorescence intensity of the whole body was significantly reduced along with time compared with the control with post‐injection with PBS. Even just after 10 min, the effect was remarkable, which could be a result of the fast IEDDA reaction between **PS‐Tz** and **BCN‐Q**. This feature is highly desirable for antidotes.

To quantitatively evaluate the deactivation efficiency, the fluorescence intensity per unit area of the skin at the backside was determined at different time points over a period of 24 h after the injection of **BCN‐Q** or PBS at 24 h post‐injection of **PS‐Tz**. As shown in Figure 5b, while the fluorescence intensity was slightly increased with time upon intravenous injection of PBS, the intensity was rapidly and significantly decreased when **BCN‐Q** was subsequently injected. At 24 h post‐administration of **BCN‐Q**, the mice were sacrificed. The skin tissues from different positions, namely the leg, back, and belly, as well as some major organs, including the spleen, lung, liver, kidney, and heart were then harvested and imaged. As shown in Figure 5c–f, the fluorescence intensities of all the skin tissues and organs of the **BCN‐Q**‐treated mice were significantly weaker than those of the PBS‐treated control, which were in accordance with the whole‐body imaging results. For the skin tissues, the reduction in fluorescence intensity was in the range of 55–72%. These results clearly demonstrated that **BCN‐Q** could effectively deactivate **PS‐Tz** in vivo, reducing its fluorescence in the skin and major organs.

The in vivo deactivation via bioorthogonal coupling was further demonstrated using liquid chromatography – mass spectrometry (LC‐MS). After the intravenous injection of **PS‐Tz** and **BCN‐Q** as mentioned above, blood samples were collected from the mice at 10 min and 8 h after the injection of **BCN‐Q**, which were then subject to LC‐MS analysis. Figure S12a (Supporting Information) shows the HPLC chromatogram of a blood sample collected at 10 min post‐injection. Apart from the signals due to **PS‐Tz** and **BCN‐Q**, an intense signal with a retention time of 28.7 min was also observed. Its ESI mass spectrum showed the base peak at *m*/*z* 872 and another signal at *m*/*z* 1742, which could be assigned to the [M+H]^2+^ and [M]^+^ ions of the clicked product **PS‐Q**, respectively (Figure S12b, Supporting Information). For the sample collected at 8 h post‐injection of **BCN‐Q**, the presence of **PS‐Q** was also confirmed by LC‐MS analysis (Figure S12c,d, Supporting Information). In the HPLC chromatogram, the signal due to **PS‐Tz** was almost invisible, showing that the click reaction had already been completed in the blood within 8 h.

### 2.6 In Vivo PDT Followed by Bioorthogonal Deactivation

Before the study of in vivo PDT using **PS‐Tz**, its biodistribution in HT29 tumor‐bearing nude mice after intravenous injection (20 nmol, 100 µL) was first monitored over a period of 48 h using the aforementioned infrared imaging system. As shown in Figure S13a (Supporting Information), the fluorescence intensity in the body of the mice grew gradually along with time. At the tumor site, the fluorescence intensity was also increased with time and almost reached the maximum after 24 h (Figure S13b, Supporting Information). Ex vivo study at 48 h post‐injection showed that the fluorescence intensity per unit area in the tumor was comparable with, if not higher than, that in some major organs (Figure S13c,d, Supporting Information). These results showed that **PS‐Tz** can accumulate in tumor along with time, which enables it to be used for photodynamic elimination of tumor.

**Figure 6:**
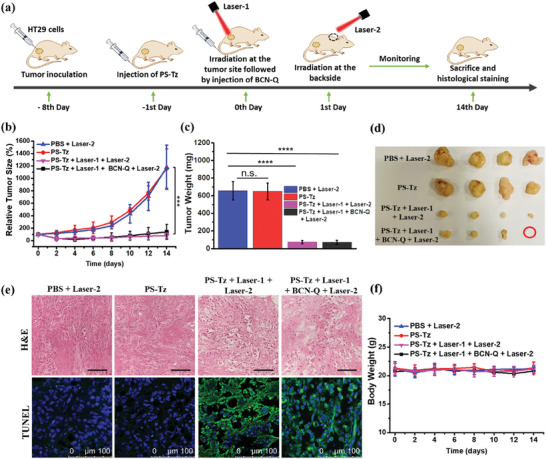
a) Timeline for studying the PDT efficacy of **PS‐Tz** in HT29 tumor‐bearing nude mice and the bioorthogonal deactivation effect of **BCN‐Q** after the photodynamic treatment. b) Tumor growth curves for HT29 tumor‐bearing nude mice after different treatments: 1) intravenous injection with PBS with the treatment of Laser‐2, 2) intravenous injection with **PS‐Tz** without laser irradiation, 3) intravenous injection with **PS‐Tz** followed by the treatment of Laser‐1 and Laser‐2, and 4) intravenous injection with **PS‐Tz** with the treatment of Laser‐1, followed by intravenous injection with **BCN‐Q** with the treatment of Laser‐2 (*n* = 4 for each group). Drug dose: 100 µL of **PS‐Tz** (20 nmol) in PBS in the presence of 0.1% Tween 80 (v/v); 200 µL of **BCN‐Q** (40 nmol) in PBS. Laser‐1: 675 nm, 0.3 W, 180 J cm^−2^; Laser‐2: 675 nm, 0.6 W, 360 J cm^−2^. c) Weights and d) photographs of the tumors in the mice harvested on Day 14 after the above treatments. e) H&E and TUNEL‐stained images of the tumor slices of the mice after the above treatments. Scale bar: 50 µm for the H&E‐stained images. The apoptotic cells and the cell nucleus were stained by TUNEL (green) and Hoechst 33342 (blue), respectively. f) Variation of the body weight of the mice receiving the above treatments over a period of 14 days. For (b), (c), and (f), data are reported as the mean ± SD of four mice in each group. n.s., not significant; ***p ≤ 0.001; ****p ≤ 0.0001.

The PDT efficacy of **PS‐Tz** in HT29 tumor‐bearing nude mice and the bioorthogonal deactivation effect of **BCN‐Q** after the photodynamic treatment were then investigated according to the procedure outlined in **Figure** **6**a. In short, HT29 cells were inoculated subcutaneously on the back of the mice. When the tumor volume reached a size of ≈60 mm^3^, the mice were injected intravenously with **PS‐Tz** (20 nmol). After 24 h when the accumulation of this compound in the tumor almost reached the maximum as shown in Figure S13b (Supporting Information), the tumor was irradiated with a diode laser at 675 nm operated at 0.3 W for 10 min (total fluence = 180 J cm^−2^) (labeled as Laser‐1) to initiate the photodynamic treatment. The mice were then injected intravenously with **BCN‐Q** (40 nmol). After further 24 h at which virtually all the **PS‐Tz** had been quenched by **BCN‐Q** as shown in Figure 5b, the backside of the mice was irradiated with another laser (675 nm, 0.6 W, 360 J cm^−2^, labeled as Laser‐2) to study the bioorthogonal deactivation effect of **BCN‐Q**. As the fluorescence intensity per unit area of the tumor was higher than that of the skin as shown in Figure S13a (Supporting Information), we used a stronger light dose arbitrary set as 2‐fold to ensure that its effect on the skin is substantial and should be at least as strong as that on the tumor. The mice were monitored for a period of 14 days after the injection of **BCN‐Q**, and then they were sacrificed for detailed analysis using various controls.

We first focused on the in vivo PDT efficacy of **PS‐Tz**. Figure 6b shows the tumor growth curves for the treatment group mentioned above and various controls. It can be seen that for the negative control of intravenous injection of PBS without applying Laser‐1 at the tumor, the tumor grew continuously over 14 days. A similar situation was observed when the mice were just injected with **PS‐Tz** without the laser treatment. In contrast, when the mice were treated with **PS‐Tz** followed by the treatment of Laser‐1, the growth of the tumor was greatly inhibited, regardless of whether **BCN‐Q** and Laser‐2 were applied, showing that **PS‐Tz** exhibited a strong antitumor PDT effect, and the post‐treatment of **BCN‐Q** and Laser‐2 did not affect its PDT efficacy. Figure S14 (Supporting Information) shows the representative images of the mice before and after the different treatments. For the groups receiving the treatment of Laser‐1, a scar could clearly be seen at the tumor which faded out gradually. It should be caused by the photodynamic effect of **PS‐Tz** instead of the laser irradiation as we confirmed previously^[^ ^30d^ ^]^ and below.

On day 14 after the various treatments, the mice were sacrificed and the tumors were harvested. Both the weight and the size of the tumors were significantly higher/larger for the PBS and **PS‐Tz** control groups compared with the other two groups receiving the treatment of **PS‐Tz** and Laser‐1 (Figure 6c,d). These results were fully consistent with the tumor growth curves shown in Figure 6b. The tumor slides were then subjected to hematoxylin and eosin (H&E) staining for histological analysis. As shown in Figure 6e (upper row), while no abnormality was observed for the PBS and **PS‐Tz** control groups, notable necrotic and apoptotic cells were detected in the slides of the tumors being treated with **PS‐Tz** and Laser‐1, reflecting a high degree of photodamage. These tumor slides were further examined using the terminal deoxynucleotidyl transferase dUTP nick‐end labeling (TUNEL) assay (Figure 6e, lower row). Bright green fluorescence was observed only in the tumor tissues from the mice receiving the photodynamic treatment of **PS‐Tz**, which indicated the occurrence of extensive cell apoptosis. For the PBS and **PS‐Tz** without the treatment of Laser‐1 control groups, the fluorescence was not noticeable, showing that the tumor remained essentially intact.

To examine the general toxicity of these treatments, we also harvested the heart, kidneys, liver, lung, and spleen of the mice on Day 14 and performed H&E staining. It was found that there was no toxicological lesion or major abnormality in the stained organ slides (Figure S15, Supporting Information), showing that all these treatments did not induce significant toxicity to these internal organs. We also monitored the body weight of the mice for each treatment group over a period of 14 days (Figure 6f). The negligible change also suggested that the treatments did not cause notable adverse effects to the mice.

**Figure 7:**
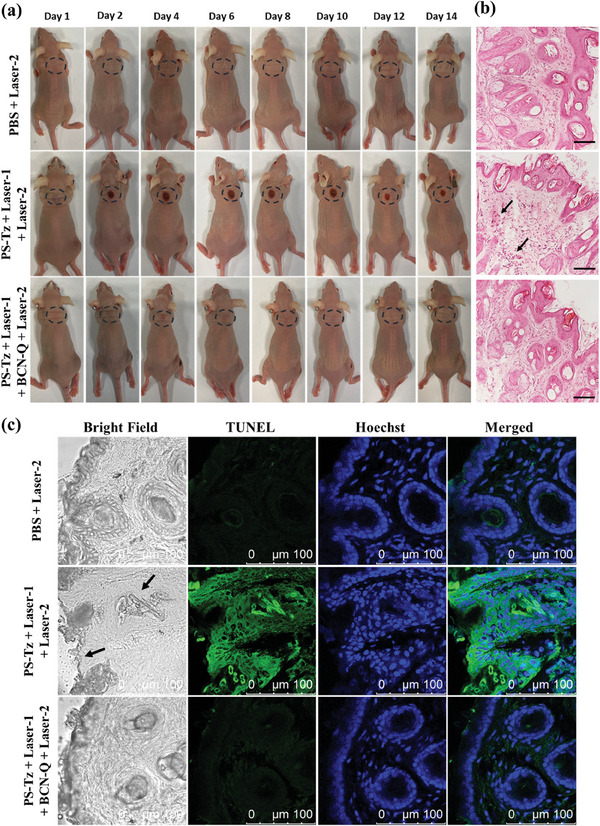
a) Photographs of one of the mice of each of the groups 1, 3, and 4 as described above over a period of 14 days. The irradiated sites of Laser‐2 are indicated with ovals. b) H&E‐stained images of the skin tissues of the mice after the above treatments. Scale bar: 100 µm. c) TUNEL‐stained images of the skin tissues of the mice after the above treatments. The apoptotic cells and the cell nucleus were stained by TUNEL (green) and Hoechst 33342 (blue), respectively.

As photosensitivity is a very common side effect of PDT, the deactivating effect of the antidote **BCN‐Q** was focused on the skin tissues. We first monitored the mice of groups (1), (3), and (4) as described above, for which Laser‐2 was applied, over a period of 14 days from the administration of **BCN‐Q**. **Figure** **7**a shows the photographs of one of the mice in each group, while those of the other three mice in each group are given in Figures S16–S18 (Supporting Information). It can be seen that the results were highly consistent. For the PBS‐treated mice (i.e., group 1), no noticeable photosensitivity was observed at the back of the mice, confirming that this power of laser irradiation would not cause significant damage to the skin. In contrast, the mice being sequentially treated with **PS‐Tz**, Laser‐1, and Laser‐2 (i.e., group 3) suffered from severe skin erythema and edema at the irradiated site of Laser‐2. The condition was not significantly improved over 14 days. However, when **BCN‐Q** was administered after the PDT treatment (i.e., group 4), only slight erythema was observed at the irradiated site, which recovered in a few days.

The different extent of skin damage was further validated using H&E and TUNEL staining of the corresponding skin tissues. For the PBS‐treated mice, the H&E‐stained skin tissues remained connected and did not show obvious pathological changes (Figure 7b). A similar result was observed in the skin tissues from the mice receiving post‐treatment of **BCN‐Q** after the photodynamic treatment of **PS‐Tz**. Without the post‐treatment, apoptotic keratinocytes (as indicated by black arrows) and vacuoles could clearly be seen in the tissues. The former is smaller than the healthy keratinocytes and is characterized by the dark and condensed nucleic chromatin. For the TUNEL‐stained slides (Figure 7c), damaged epidermis and hair follicles were only observed in the bright field images (as indicated by black arrows) for the mice of group 3 accompanied with strong green fluorescence appeared after the TUNEL staining, while minimal skin damage and green signal were observed for the control groups 1 and 4. All these results demonstrated that the photodynamic effect of **PS‐Tz** caused severe damage to the skin tissues, and **BCN‐Q** could largely reduce the photosensitivity induced by this photosensitizer in mice.

## 3 Conclusion

In summary, we have developed a novel and unprecedented bioorthogonal strategy for addressing the problem of photosensitivity induced after PDT. It involves the use of the antidote **BCN‐Q**, which contains a BCN moiety that can rapidly click with the tetrazine substituent of the photosensitizer **PS‐Tz** and a BHQ‐3 quencher that can effectively quench the fluorescence emission and ROS generation of **PS‐Tz** after the covalent linkage through a FRET process. The efficient quenching of these photoactivities has been demonstrated in PBS and a range of cancer cell lines using various controls. Using nude mice as an animal model, it has also been shown that the injection of this antidote can effectively deactivate the residual photosensitizer in the body of the mice without affecting the PDT efficacy. In particular, the skin tissues showed minimal damage after the post‐treatment with **BCN‐Q** after the PDT with **PS‐Tz** as shown by the physical appearance of the skin as well as the H&E and TUNEL staining. Compared with the approach of using activatable photosensitizers to enhance the tumor specificity and reduce the unwanted photodamage to non‐target sites, this highly efficient bioorthogonal deactivating strategy is not affected by the subtle difference in the levels of the corresponding stimuli in cancer and normal cells. Its straightforward “click‐and‐quench” mechanism ensures the residual photosensitizer in the body can be deactivated effectively after the photodynamic treatment. The overall results demonstrate that this bioorthogonal approach can effectively suppress the photosensitivity after PDT in nude mice and would be potentially useful in minimizing this common side effect of PDT, thereby promoting its clinical application.

## 4 Experimental Section

### General

All reactions were performed under an atmosphere of nitrogen. DMF and CH_2_Cl_2_ were dried using an INERT solvent purification system. PBS at pH 7.4 was used in all the studies. All other solvents and reagents were used as received. Chromatographic purification was performed on silica gel (Macherey‐Nagel 230–400 mesh) with the indicated eluent. Compounds **1**,^[^ ^24^ ^]^ **2**,^[^ ^25^ ^]^ **PS‐N_3_**,^[^ ^32^ ^]^ and **BCN‐Q** ^[^ ^30d^ ^]^ were prepared according to the literature procedure.

### Instrumentation

^1^H and ^13^C{^1^H} NMR spectra were recorded on a Bruker AVANCE III 400 MHz spectrometer or a Bruker AVANCE III 500 MHz spectrometer in CDCl_3_. Spectra were referenced internally by using the residual solvent (^1^H: δ 7.26) or solvent (^13^C: δ 77.2) resonance relative to SiMe_4_. ESI mass spectra were recorded on a Thermo Finnigan MAT 95 XL mass spectrometer. Electronic absorption and steady‐state fluorescence spectra were taken on a Cary 5G UV–Vis–NIR spectrophotometer and a HORIBA FluoroMax‐4 spectrofluorometer, respectively. Reverse‐phase HPLC separation was performed on an Apollo‐C18 column (5 µm, 4.6 mm × 150 mm) at a flow rate of 1 mL min^−1^ for analytical purpose or on a XBridge BEH300 Prep C18 column (5 µm, 10 mm × 250 mm) at a flow rate of 3 mL min^−1^ for preparative purpose, using a Waters system equipped with a Waters 1525 binary pump and a Waters 2998 photodiode array detector. The solvents used for HPLC analysis were of HPLC grade. LC‐MS studies were performed on a XSelect CSH C18 column (5 µm, 4.6 mm × 250 mm) at a flow rate of 0.8 mL min^−1^ using a Waters system equipped with a Waters Quaternary Solvent Manager‐R, a Waters 2998 photodiode array detector, a Waters 2475 fluorescence detector, and a Waters single quadrupole detector 2. The solvents used were of LC‐MS grade.

### Preparation of PS‐Tz

BODIPY **1** (120 mg, 0.21 mmol) and benzaldehyde **2** (224 mg, 0.84 mmol) were dissolved in toluene (50 mL). Acetic acid (0.3 mL) and piperidine (0.3 mL) were then added. The mixture was heated under reflux with a Dean–Stark trap. After the consumption of **1** as indicated by thin‐layer chromatography, the mixture was cooled to room temperature, and the solvent was evaporated under reduced pressure. Water (150 mL) was then added to the residue, and the crude product was extracted with CH_2_Cl_2_ (100 mL × 3). The combined organic phase was dried over anhydrous Na_2_SO_4_, and the solvent was evaporated under reduced pressure. The crude product was purified by column chromatography on silica gel with CH_2_Cl_2_/CH_3_OH (70:1 v/v) as eluent to afford **PS‐Tz** as a green solid (85 mg, 38%). ^1^H NMR (400 MHz, CDCl_3_): δ 8.76 (d, *J* = 8.4 Hz, 2 H, ArH), 7.57‐7.64 (m, 8 H, ArH and C=CH), 6.95 (d, *J* = 8.8 Hz, 4 H, ArH), 6.64 (virtual s, 2 H, ArH), 4.19 (t, *J* = 4.8 Hz, 4 H, OCH_2_), 3.89 (t, *J* = 4.8 Hz, 4 H, OCH_2_), 3.75‐3.78 (m, 4 H, OCH_2_), 3.66‐3.72 (m, 8 H, OCH_2_), 3.55‐3.58 (m, 4 H, OCH_2_), 3.39 (s, 6 H, OCH_3_), 3.14 (s, 3 H, Tz‐CH_3_), 1.51 (s, 6 H, CH_3_). ^13^C{^1^H} NMR (125.8 MHz, CDCl_3_): δ 167.7, 160.1, 148.8, 140.6, 139.4, 139.3, 129.8, 129.7, 129.4, 128.7, 115.0, 71.9, 70.9, 70.7, 70.6, 69.7, 67.5, 59.1, 29.7, 21.3 14.0. HRMS (ESI): *m/z* calcd for C_50_H_53_BBr_2_F_2_N_6_O_8_ [M‐H]^−^, 1076.2509; found, 1076.2495.

### Preparation of **PS‐Q**

**PS‐Tz** (2.0 mg, 1.86 µmol) and **BCN‐Q** (2.0 mg, 2.88 µmol) were dissolved in CH_3_CN (1 mL). The mixture was stirred at room temperature for 2 h, followed by purification by HPLC. The conditions were set as follows: solvent A = 0.1% trifluoroacetic acid (TFA) in CH_3_CN; solvent B = 0.1% TFA in distilled water. The gradient was 20% A + 80% B in the first 5 min, changed to 100% A in 30 min, maintained under this condition for 10 min, changed to 100% B in 5 min, and then kept at this condition for 10 min. The conjugate was obtained as a dark bluish green solid (3.1 mg, 95%). HRMS (ESI): *m*/*z* calcd for C_93_H_103_BBr_2_F_2_N_11_O_10_ [M]^+^, 1742.6321; found, 1742.6313.

### Bioorthogonal Quenching in Solution

The deactivation of **PS‐Tz** and **PS‐N_3_** by **BCN‐Q** was performed in a 1 cm × 1 cm quartz cuvette. Stock solutions of **PS‐Tz** and **PS‐N_3_** were prepared in DMF (2 mm), which were diluted to 1 µm with PBS in the presence of 0.1% Tween 80 (v/v). Another stock solution of **BCN‐Q** in DMF (2 mm) was also prepared, which was then added to the above solution of **PS‐Tz** or **PS‐N_3_** to attain the final concentration of **BCN‐Q** to 1, 2, and 3 µm, respectively. The fluorescence spectra were recorded in the range of 650–800 nm at different time points upon excitation at 610 nm. To study the bioorthogonal deactivation in ROS formation, a mixture of DPBF (30 µm) and **PS‐Q** or **PS‐Tz** (1 µm) with or without the treatment with **BCN‐Q** (1, 2, and 3 µm) for 6 min in PBS in the presence of 0.1% Tween 80 (v/v) was irradiated with red light from a 300 W halogen lamp after passing through a water tank for cooling and a color filter with a cut‐on wavelength at 610 nm (Newport). The absorption maximum of DPBF at 417 nm was monitored along with the irradiation time. For the measurements of fluorescence and singlet oxygen quantum yields, the procedures as described previously were followed.^[^ ^40^ ^]^

### Cell Lines and Culture Conditions

HT29 cells (ATCC, no. HTB‐38) were maintained in Roswell Park Memorial Institute (RPMI) 1640 medium (Invitrogen, no. 23400‐021). A549 (ATCC, no. CCL‐185) and HepG2 (ATCC, no. HB‐8065) cells were maintained in DMEM (ThermoFisher Scientific, no. 12100‐046). Both media were supplemented with FBS (10%) and a penicillin‐streptomycin solution (100 unit mL^−1^ and 100 mg mL^−1^, respectively). All the cells were grown at 37 °C in a humidified 5% CO_2_ atmosphere.

### Confocal Fluorescence Microscopic Study

Approximately 2 × 10^5^ HT29, A549, and HepG2 cells in the corresponding culture medium (2 mL) were seeded on glass‐bottom confocal dishes and incubated at 37 °C overnight in a humidified 5% CO_2_ atmosphere. After removal of the medium, the cells were rinsed with PBS and incubated in the medium with **PS**‐**Tz** (1 µm) for 6 h. After discarding the medium and rinsing the cells with PBS twice, the cells were further incubated in the medium with or without **NH_2_‐Q** or **BCN‐Q** (2 µm) for 1 h. For comparison, the cells were also incubated with the conjugate **PS‐Q** (1 µm) instead of **PS**‐**Tz** for 6 h without the post‐treatment or simply with **BCN‐Q** (2 µm) for 1 h. After being rinsed with PBS again, all the cells were replenished with Hank's Balanced Salt Solution (HBSS) (1 mL) before being examined using a Leica TCS SP8 high‐speed confocal microscope equipped with a solid‐state 638 nm laser. The photosensitizer was excited at 638 nm and its fluorescence was monitored at 650–750 nm. The images were digitized and analyzed using a Leica Application Suite X software.

### Study of Intracellular ROS Generation

HT29, A549, and HepG2 cells, which had been treated as described above, were further stained with H_2_DCFDA (50 µm) for 30 min. The cells were then rinsed with PBS for three times followed by the dark or light treatment at ambient temperature for 20 min. The light source consisted of a 300 W halogen lamp, a water tank for cooling, and a colored glass filter with a cut‐on wavelength at 610 nm (Newport). The fluence rate (λ > 610 nm) was 23 mW cm^−2^. Illumination of 20 min led to a total fluence of 28 J cm^−2^. The fluorescence of DCF in these cells was imaged using confocal fluorescence microscopy. The DCF was excited at 488 nm, and the fluorescence was monitored at 515–580 nm.

### Study of Photocytotoxicity

Approximately 1 × 10^4^ HT29, A549, and HepG2 cells per well in the corresponding culture medium were inoculated in 96‐well plates and incubated at 37 °C overnight in a humidified 5% CO_2_ atmosphere. The stock solution of **PS‐Tz** in DMF (2 mm) was diluted to various concentrations with the culture medium. The cells were then incubated with 100 µL of these drug solutions for 6 h. After being rinsed with PBS for three times, the cells were further incubated in the medium with or without two equiv. of **BCN‐Q** for 1 h. After that, these cells were rinsed again with PBS and refed with 100 µL of the culture medium before being irradiated at ambient temperature with the above light source for 20 min (total fluence = 28 J cm^−2^). Cell viability was determined by means of a colorimetric MTT assay as described previously.^[^ ^40^ ^]^

### Hemolysis Assay

The blood compatibility of **PS‐Tz**, **BCN‐Q**, and **PS‐Q** was evaluated with a hemolysis assay. Fresh rabbit blood was obtained from the Laboratory Animal Services Centre of The Chinese University of Hong Kong. A blood sample (2 mL) was centrifuged at 9000 rpm for 10 min to isolate the red blood cells (RBCs). The red pellet on the bottom was washed with PBS for three times. After that, these RBCs (400 µL) were mixed with PBS (9.6 mL) to give a RBC suspension (4% v/v). Stock solutions of **PS‐Tz**, **BCN‐Q**, and **PS‐Q** in DMF (2 mM) were diluted with PBS to various concentrations (up to 40 µm). These solutions (50 µL) were then mixed with the RBC suspension (100 µL). After incubation at 37 °C in the dark for 6 h, the mixtures were centrifuged at 1500 rpm for 5 min. Aliquots (100 µL) of the supernatant were transferred to 96‐well plates, and the hemoglobin released was monitored at 540 nm using a microplate reader (Tecan Spark 10 m). PBS and 0.5% Triton X‐100 were used as the negative and positive controls, respectively. The percentage of hemolysis was calculated using the following equation:

(1) $$\mathrm{Hemolysis}\;\%=\frac{A_{540}\;\mathrm{in}\;\mathrm{solution}-A_{540}\;\mathrm{in}\;\mathrm{PBS}}{\;A_{540}\;\mathrm{in}\;\mathrm{Triton}\;X-100-A_{540}\;\mathrm{in}\;\mathrm{PBS}}\times 100$$

### In Vivo Bioorthogonal Deactivation

Female BALB/c nude mice (20–25 g) were obtained from the Laboratory Animal Services Centre of The Chinese University of Hong Kong. All animal experiments had been approved by the Animal Experimentation Ethics Committee of the University (Ref. No. 21‐005‐GRF). The mice were kept under a pathogen‐free condition with free access to food and water. To evaluate the in vivo quenching efficiency of **BCN‐Q**, the mice were intravenously injected with **PS‐Tz** in PBS in the presence of 0.1% Tween 80 (v/v) (20 nmol, 100 µL). After being kept in the dark for 24 h, the mice were intravenously injected with **BCN‐Q** in PBS (40 nmol, 200 µL) or PBS (200 µL). The fluorescence images of the mice were captured before and after the injection at different time points up to 24 h from the injection of **BCN‐Q** or PBS, using an Odyssey infrared imaging system (excitation wavelength = 680 nm, emission wavelength ≥ 700 nm). The images were digitized and analyzed using an Odyssey imaging system software (9201‐500). After the in vivo imaging study, the animals were euthanized at 24 h post‐injection of **BCN‐Q** or PBS. The skin tissues and major organs were harvested, and their fluorescence intensities were measured. Three mice were used for each group

### LC‐MS Analysis of In Vivo Bioorthogonal Deactivation

After sequential intravenous injection of **PS‐Tz** and **BCN‐Q** as described above, blood samples (50 µL) were collected from the mice at 10 min and 8 h after the injection of **BCN‐Q**. MeOH (200 µL) was then added to each blood sample, followed by centrifugation at 10 000 rpm for 10 min. The supernatant was collected and analyzed by LC‐MS. The conditions were set as follows: solvent A = 0.01% formic acid in acetonitrile; solvent B = 0.01% formic acid in deionized water. The gradient was 20% A + 80% B in the first 5 min, changed to 0% A + 100% B in 30 min, maintained under this condition for 10 min, and then changed to 100% A + 0% B in 15 min.

### In Vivo Biodistribution

To evaluate the in vivo biodistribution and tumor localization property of **PS‐Tz**, HT29 cells (≈1 × 10^7^ cells in 200 µL HBSS) were inoculated subcutaneously on the back of the mice. The tumor volume was calculated using the following formula: Volume (mm^3^) = (Length × Width^2^)/2. Once the tumors had grown to a size of ≈100 mm^3^, a solution of **PS‐Tz** in PBS in the presence of 0.1% Tween 80 (v/v) (20 nmol, 100 µL) was injected into the tumor‐bearing mice through tail intravenous injection. The fluorescence images of the mice were captured before and after the injection at different time points up to 48 h using the imaging system mentioned above. After the in vivo imaging study, the animals were euthanized at 48 h post‐injection. The tumors and major organs were harvested, and their fluorescence intensities were measured. Four mice were used for each group.

### In Vivo PDT Followed by Bioorthogonal Deactivation

HT29 cells (≈1 × 10^7^ cells in 200 µL HBSS) were inoculated subcutaneously on the back of the mice. The treatment started when the tumor volume reached ≈60 mm^3^. The HT29 tumor‐bearing mice were randomly divided into four groups: 1) intravenous injection with PBS with the treatment of Laser‐2, 2) intravenous injection with **PS‐Tz** without laser irradiation, 3) intravenous injection with **PS‐Tz** followed by the treatment of Laser‐1 and Laser‐2, and 4) intravenous injection with **PS‐Tz** with the treatment of Laser‐1, followed by intravenous injection with **BCN‐Q** with the treatment of Laser‐2. For the positive treatment group, **PS‐Tz** in PBS in the presence of 0.1% Tween 80 (v/v) (20 nmol, 100 µL) was injected intravenously into the tumor‐bearing mice. At 24 h post‐injection, the tumor was irradiated with a diode laser (Biolitec Ceralas) at 675 nm operated at 0.3 W (labeled as Laser‐1). Illumination on a spot size of 1.0 cm^2^ for 10 min led to a total fluence of 180 J cm^−2^. After that, the mice were further received an intravenous dose of **BCN‐Q** in PBS (40 nmol, 200 µL). After further 24 h, the backside of the mice was irradiated with the same laser but with a different power (675 nm, 0.6 W, 360 J cm^−2^, labeled as Laser‐2). The tumor size and the irradiated area of the nude mice were monitored periodically for the next 14 days. The tumor volumes were compared with those of the control groups.

### Ex Vivo Histological Staining

After 14 days of the treatments, the mice were sacrificed, and the tumors, skin tissues, and major organs were harvested. All the tissues were then subjected to H&E and/or TUNEL staining according to the previously described procedure.^[30d]^

### Statistical Analysis

The data shown in Figure 6b,c are reported as the mean ± SD and were analyzed using the Student's *t*‐test with *p* values < 0.05 considered as significant; ***p ≤ 0.001; ****p ≤ 0.0001. Statistical calculations were performed using a GraphPad Prism software (version 8; San Diego, CA, USA).

## Conflict of Interest

The authors declare no conflict of interest.

## Supporting information

Supporting Information

## Data Availability

The data that support the findings of this study are available from the corresponding author upon reasonable request.
