# Supplementary material for: A Bioorthogonal Antidote Against the Photosensitivity after Photodynamic Therapy
Source: Adv Sci (Weinh). 2023 Dec 31;11(11):2306207. doi: 10.1002/advs.202306207 (PMC10953549; doi:10.1002/advs.202306207)
Supplement: Supplementary file 1 — Supporting Information [file ADVS-11-2306207-s001.pdf]

ADVANCED SCIENCE
Open Access

Supporting Information

for *Adv. Sci.*, DOI 10.1002/advs.202306207

A Bioorthogonal Antidote Against the Photosensitivity after Photodynamic Therapy

*Evelyn Y. Xue, Caixia Yang, Yimin Zhou and Dennis K. P. Ng**

Supporting Information

A Bioorthogonal Antidote Against the Photosensitivity after Photodynamic Therapy

*Evelyn Y. Xue, Caixia Yang, Yimin Zhou, and Dennis K. P. Ng**

E. Y. Xue, C. Yang, Y. Zhou, D. K. P. Ng

Department of Chemistry

The Chinese University of Hong Kong

Shatin, N.T., Hong Kong (China)

E-mail: dkpn@cuhk.edu.hk

<u>**Contents**</u>

Scheme S1 Synthesis of **PS-Tz**.

Figure S1 ^{1}H and $^{13}C\{^{1}H\}$ NMR spectra of **PS-Tz** in $CDCl_3$.

Figure S2 ESI mass spectrum of **PS-Tz**.

Figure S3 HPLC chromatograms of **BCN-Q** in (a) PBS and (b) DMEM with FBS (10%) recorded at different time points over a period of 14 days at room temperature.

Figure S4 (a-f) Change in the fluorescence spectrum of **PS-Tz** (1 μM) (λ_{ex} = 610 nm) in PBS in the presence of 0.1% Tween 80 (v/v) with or without the presence of different concentrations of **BCN-Q** (1, 2, and 3 μM), **NH_2-Q** (3 μM), or BCN **3** (3 μM) over a period of 24 min. (g) Molecular structure of **NH_2-Q**. (h) Molecular structure of BCN **3**.

Figure S5 (a-d) Change in the fluorescence spectrum of **PS-N_3** (1 μM) (λ_{ex} = 610 nm) in PBS in the presence of 0.1% Tween 80 (v/v) with or without the presence of different concentrations of **BCN-Q** (1, 2, and 3 μM) over a period of 24 min. (e) Variation of the fluorescence intensity at 723 nm with time under these conditions. (f) molecular structure of **PS-N_3.**

Scheme S2 Synthesis of **PS-Q**.

Figure S6 HPLC chromatogram of **PS-Q**.

Figure S7 ESI mass spectrum of **PS-Q**.

Figure S8 (a) Fluorescence spectra of **PS-Tz**, **PS-Q**, and **BCN-Q** in DMF (λ_{ex} = 610 nm). (b) Spectral overlap between the fluorescence spectrum of **PS-Tz** and the

electronic absorption spectrum of **BCN-Q** in DMF. (b) Electronic absorption spectra of **PS-Q**, **PS-Tz**, and **BCN-Q** (all at 1 μM) in DMF.

Figure 9 (a) Comparison of the rates of decay of DPBF in DMF (initial concentration = 30 μM), as monitored spectroscopically at 417 nm, using **PS-Q**, **PS-Tz**, and ZnPc (all at 1 μM) as the photosensitizers. (b) Comparison of the rates of decay of ABDA in water with 0.1% Tween 80 (v/v) (initial concentration = 30 μM), as monitored spectroscopically at 400 nm, using **PS-Q**, **PS-Tz**, and MB (all at 1 μM) as the photosensitizers.

Figure S10 Cytotoxicity of **PS-Q** against (a) HT29, (b) A549, and (c) HepG2 cells in the absence and presence of light irradiation ($\lambda > 610$ nm, 23 mW cm^{-2}) for 20 min. (d) Dark cytotoxicity of **BCN-Q** against HT29, A549, and HepG2 cells. Data are reported as the mean ± SEM of three independent experiments, each performed in quadruplicate.

Figure S11 Hemolytic activity of the photosensitizer **PS-Tz**, the antidote **BCN-Q**, and the conjugate **PS-Q** against rabbit red blood cells after incubation at 37 °C for 6 h.

Figure S12 HPLC chromatograms of the blood samples collected at (a) 10 min and (c) 8 h post-injection of **BCN-Q**. The mice were first intravenously injected with **PS-Tz** in PBS in the presence of 0.1% Tween 80 (v/v) (20 nmol, 100 μL), followed by intravenous injection of **BCN-Q** in PBS (40 nmol, 200 μL) at 24 h post-injection of **PS-Tz**. (b,d) ESI mass spectra of the corresponding fractions with a retention time of 28.7 min.

Figure S13 (a) Near-infrared ($\lambda \geq 700$ nm) fluorescence images of HT29 tumor-bearing

nude mice after intravenous injection with **PS-Tz** in PBS in the presence of 0.1% Tween 80 (v/v) (20 nmol, 100 μL) over a period of 48 h. (b) Change in fluorescence intensity per unit area of the tumor in the **PS-Tz**-treated mice along with time. (c) Ex vivo images and (d) quantified fluorescence intensities of the tumor and some major organs harvested from the mice at 48 h post-injection of **PS-Tz**.

Figure S14 Representative photographs of the mice before and after different treatments.

Figure S15 H&E-stained images of different organ slides from the mice scarified on Day 14 after different treatments.

Figure S16 Photographs of the other three mice of group 1.

Figure S17 Photographs of the other three mice of group 3.

Figure S18 Photographs of the other three mice of Group 4.

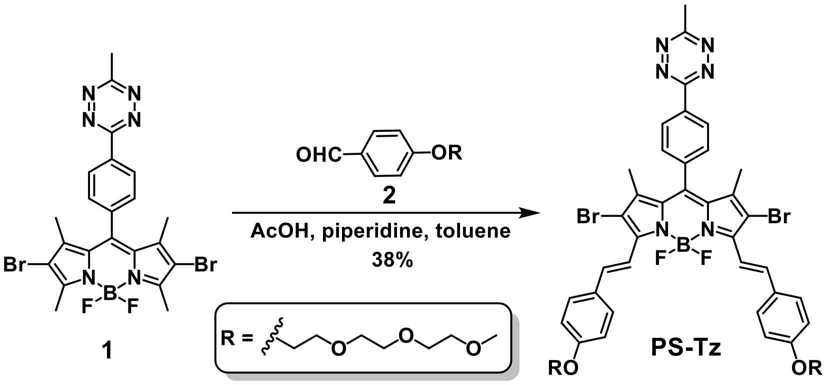

Scheme S1. Synthesis of **PS-Tz**.

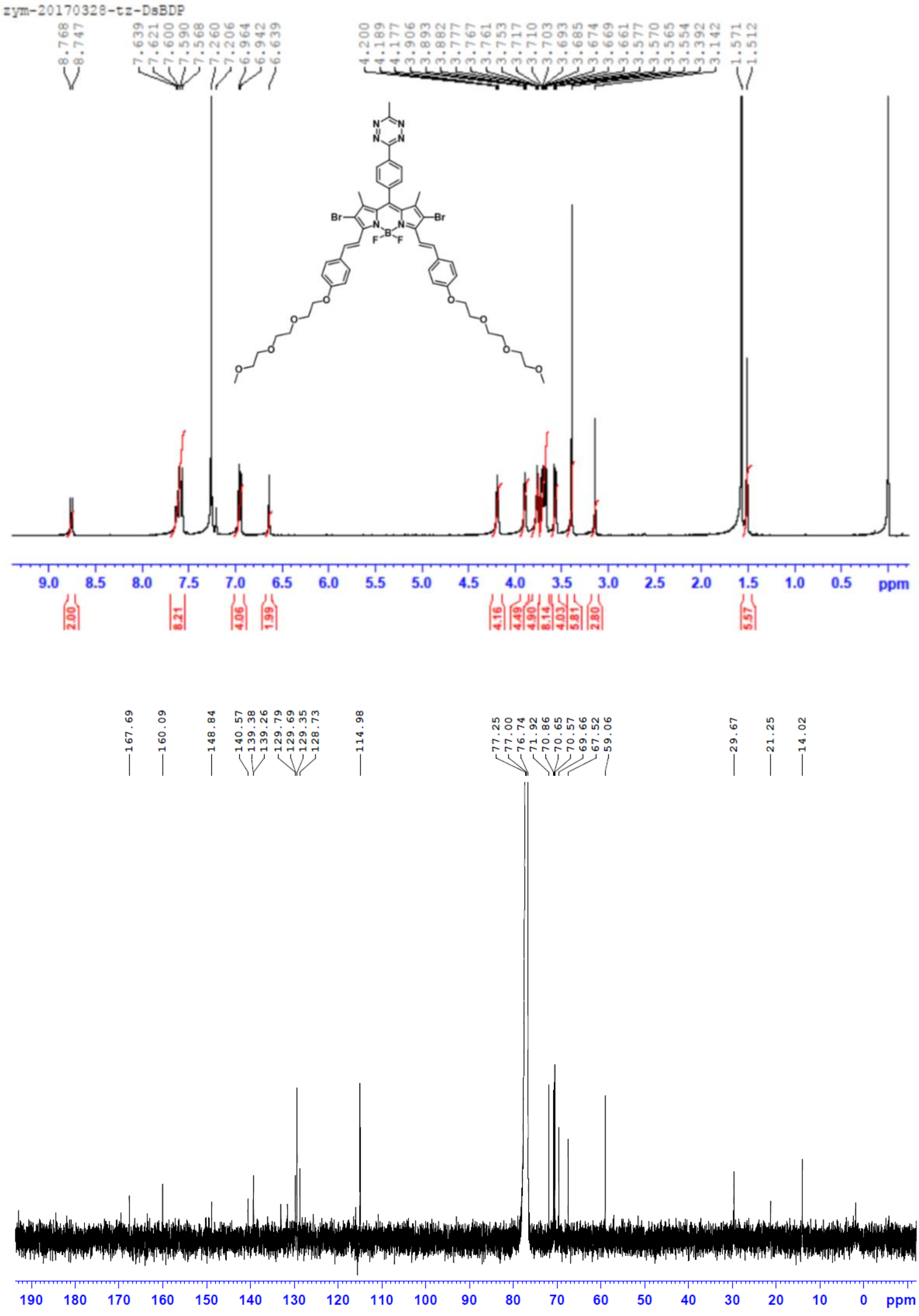

Figure S1. ^1H and $^{13}C\{^1H\}$ NMR spectra of **PS-Tz** in $CDCl_3$.

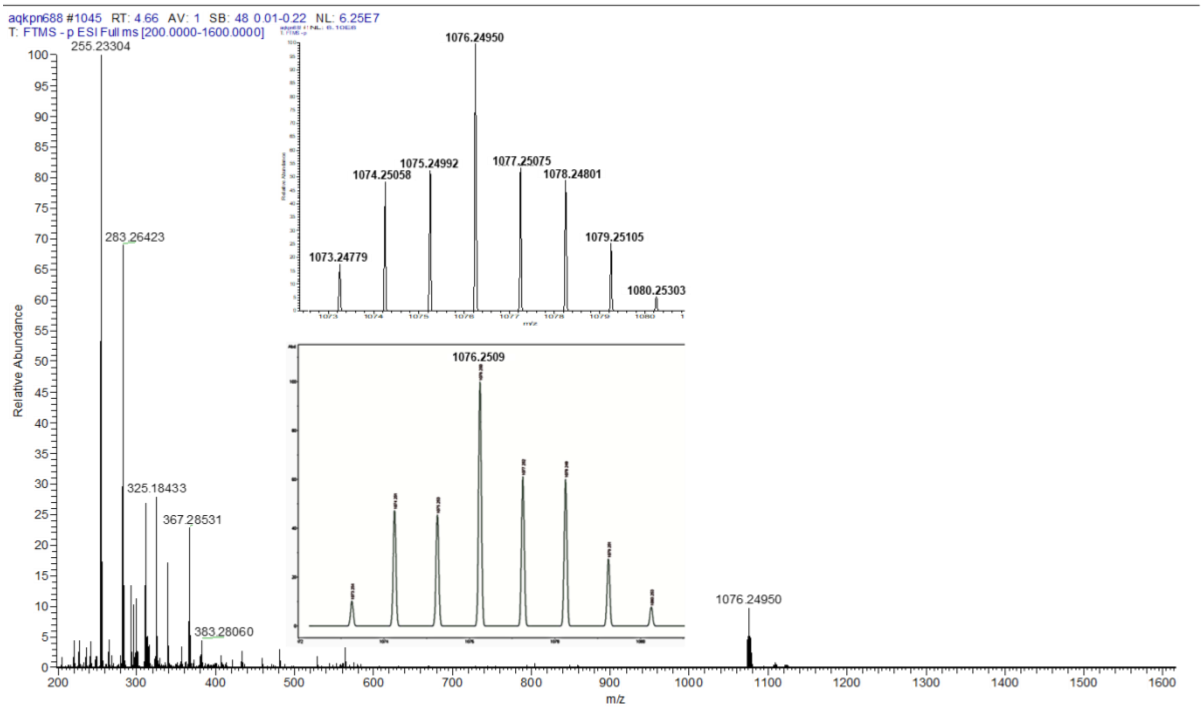

Figure S2. ESI mass spectrum of **PS-Tz**. The insets show the experimental (top) and simulated (bottom) isotopic patterns for the $[M-H]^-$ ion.

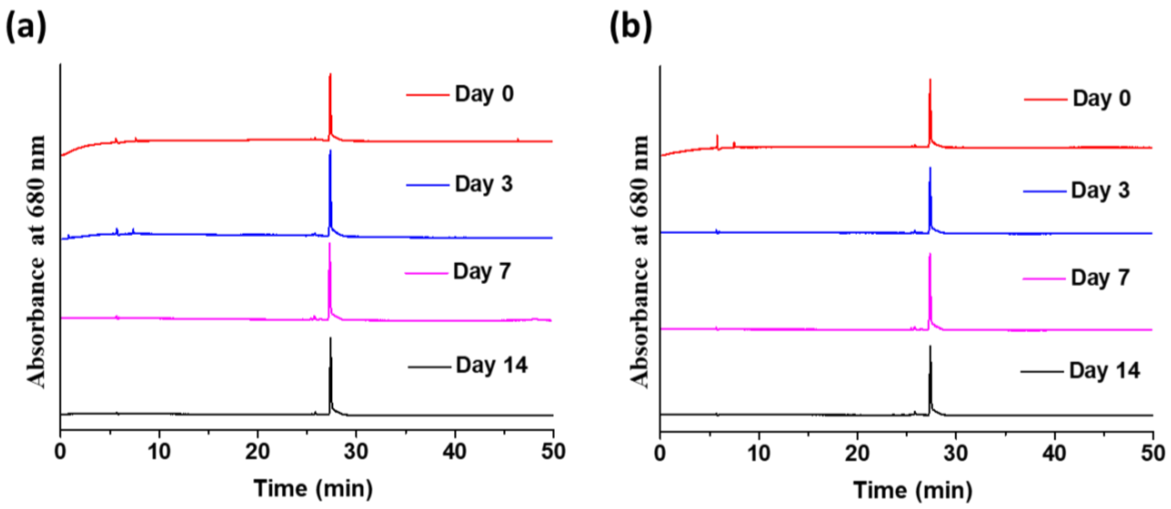

Figure S3. HPLC chromatograms of **BCN-Q** in (a) PBS and (b) DMEM with FBS (10%) recorded at different time points over a period of 14 days at room temperature.

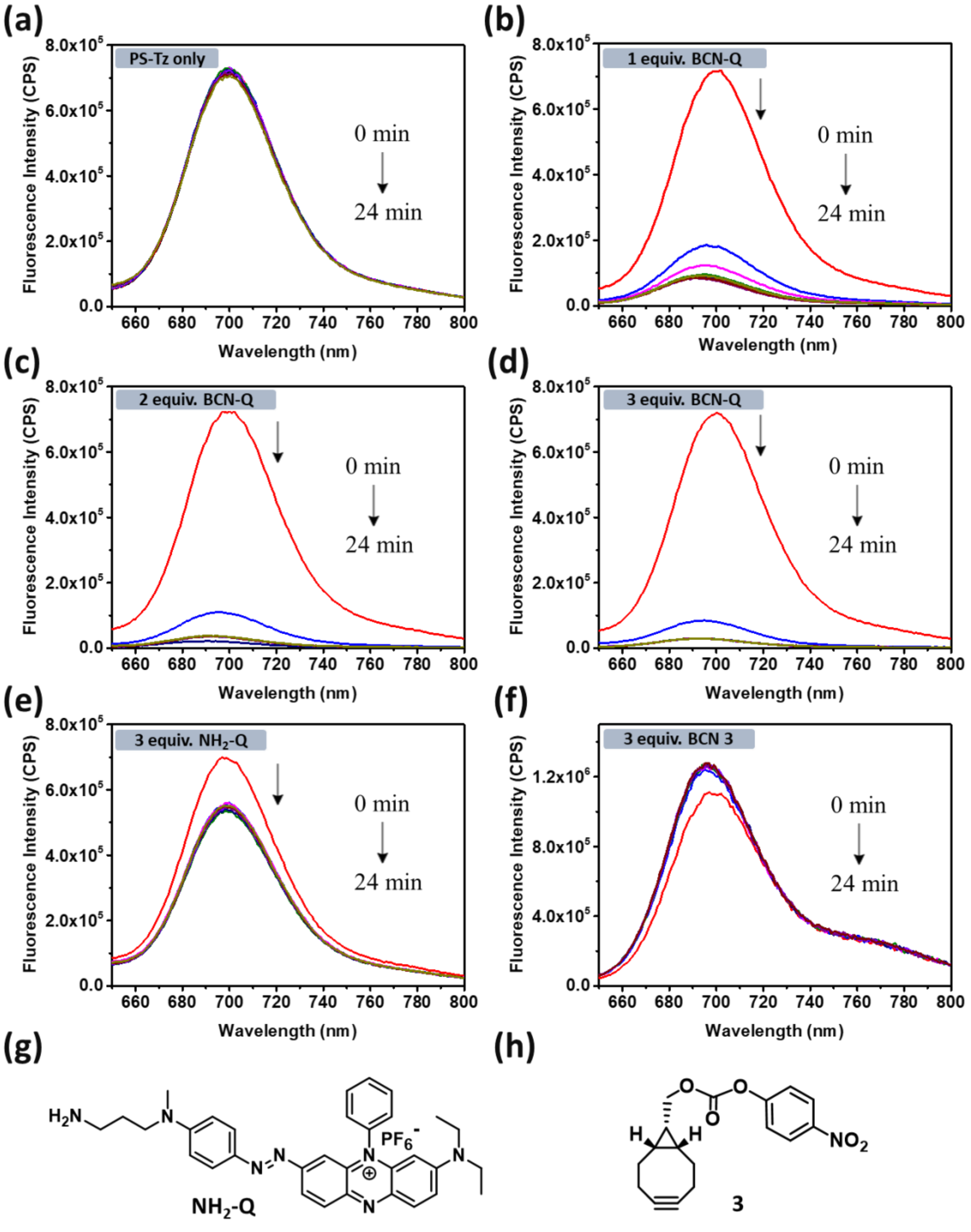

Figure S4. (a-f) Change in the fluorescence spectrum of **PS-Tz** (1 µM) (λ_{ex} = 610 nm) in PBS in the presence of 0.1% Tween 80 (v/v) with or without the presence of different concentrations of **BCN-Q** (1, 2, and 3 µM), **NH$_2$-Q** (3 µM), or BCN **3** (3 µM) over a period of 24 min. (g) Molecular structure of **NH$_2$-Q**. (h) Molecular structure of BCN **3**.

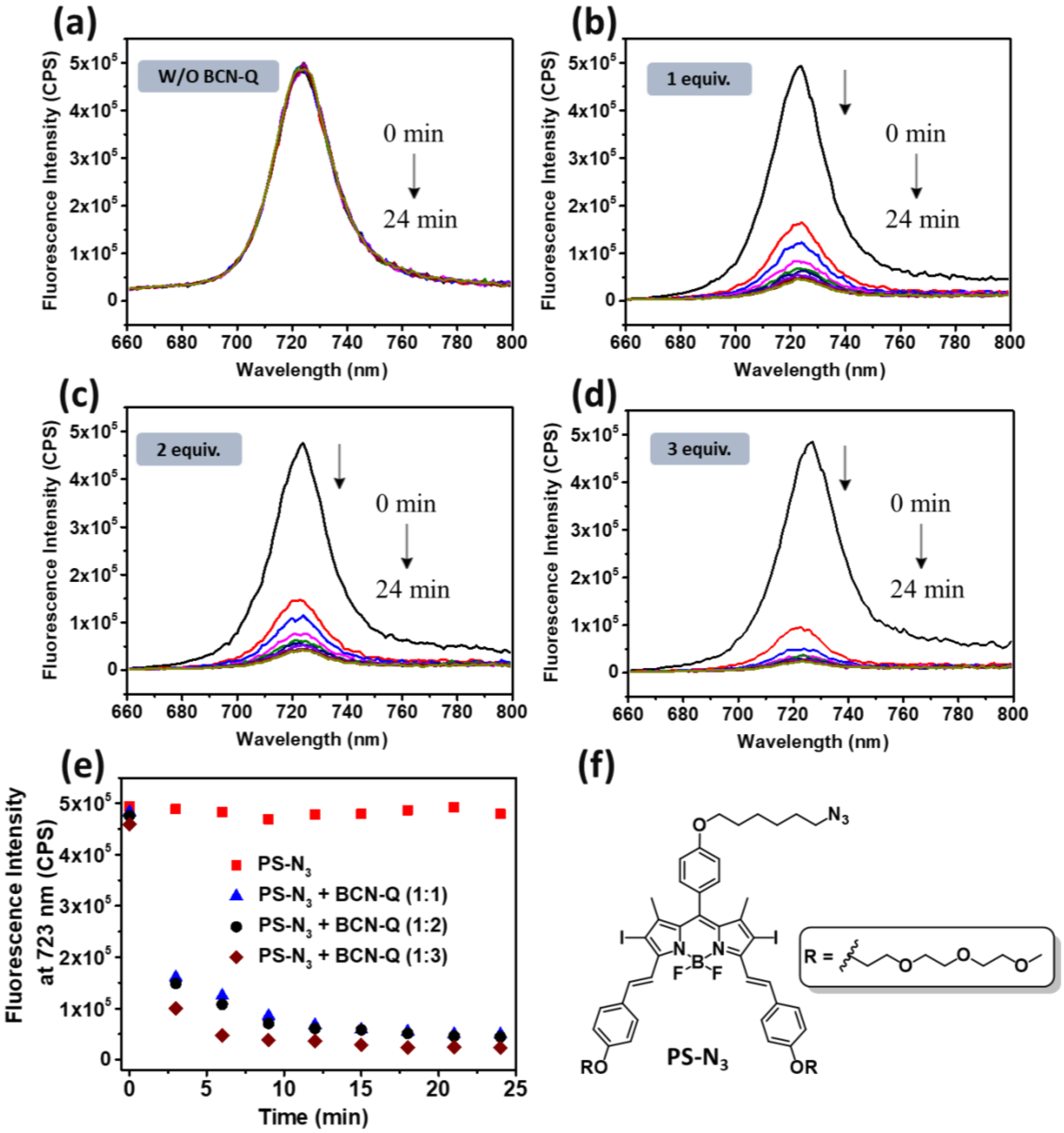

Figure S5. (a-d) Change in the fluorescence spectrum of **PS-N$_3$** (1 μM) (λ_{ex} = 610 nm) in PBS in the presence of 0.1% Tween 80 (v/v) with or without the presence of different concentrations of **BCN-Q** (1, 2, and 3 μM) over a period of 24 min. (e) Variation of the fluorescence intensity at 723 nm with time under these conditions. (f) molecular structure of **PS-N$_3$.**

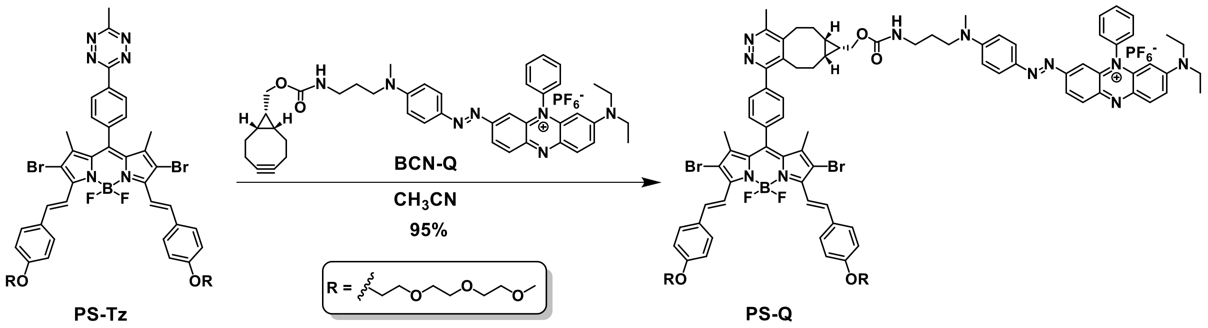

Scheme S2. Synthesis of **PS-Q**.

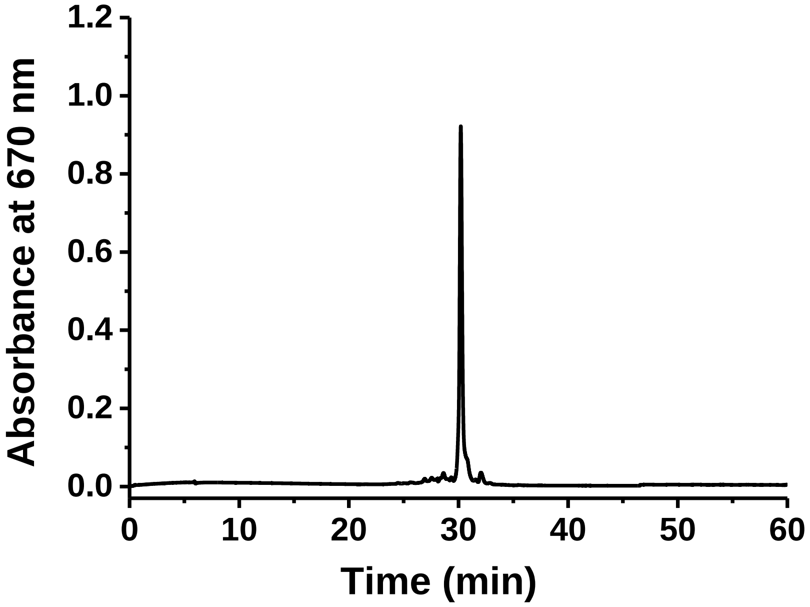

Figure S6. HPLC chromatogram of **PS-Q**.

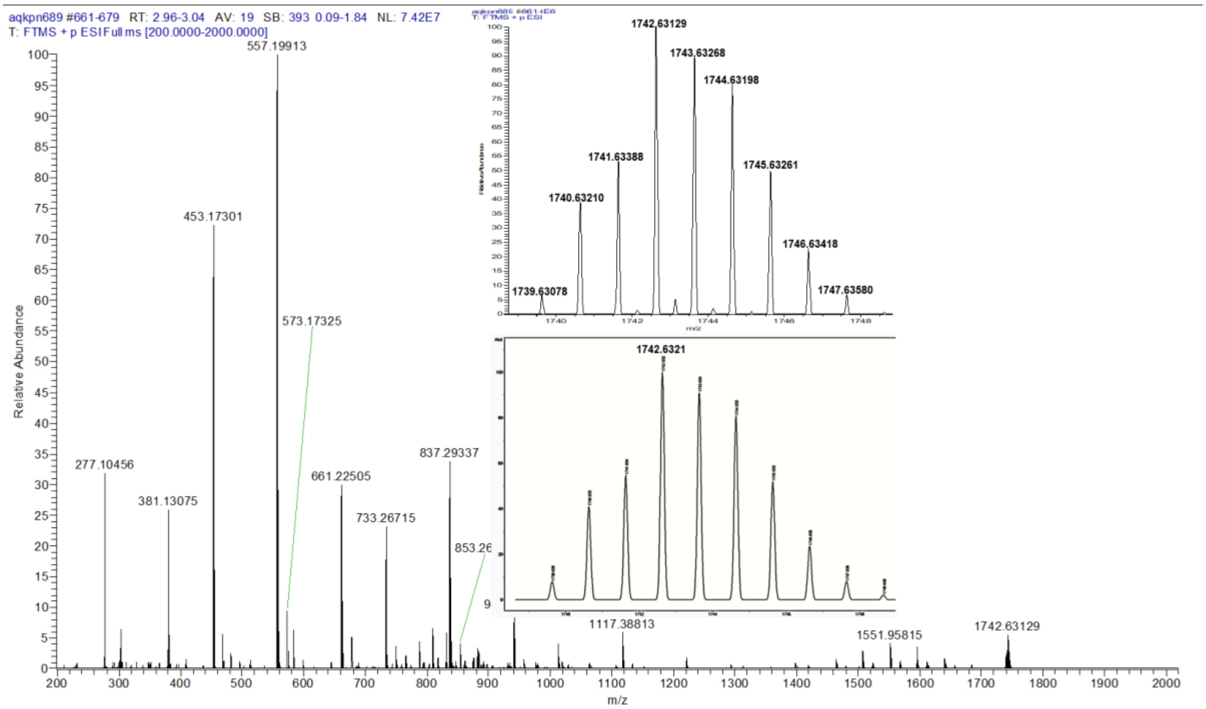

Figure S7. ESI mass spectrum of **PS-Q**. The insets show the experimental (top) and simulated (bottom) isotopic patterns for the $[M]^{+}$ ion.

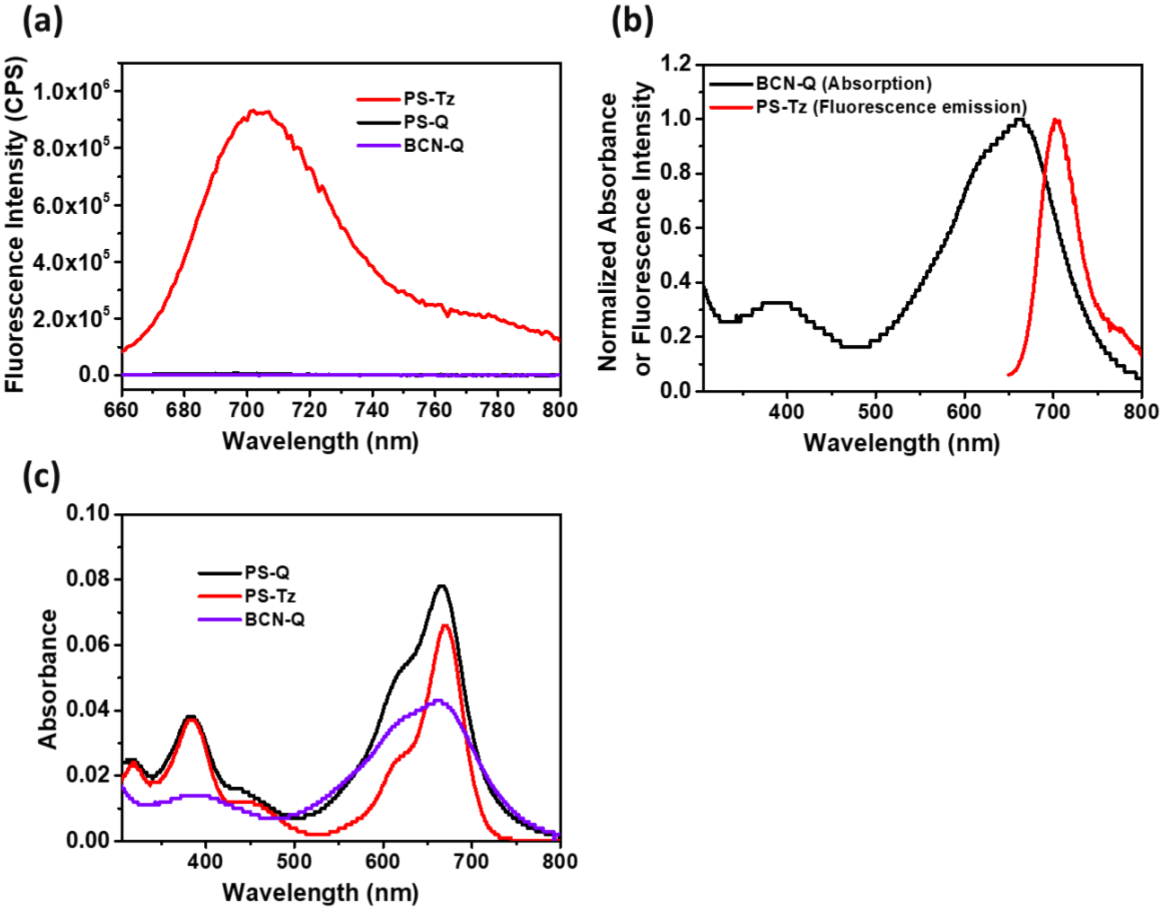

Figure S8. (a) Fluorescence spectra of **PS-Tz**, **PS-Q**, and **BCN-Q** in DMF (λ_{ex} = 610 nm). (b) Spectral overlap between the fluorescence spectrum of **PS-Tz** and the electronic absorption spectrum of **BCN-Q** in DMF. (b) Electronic absorption spectra of **PS-Q**, **PS-Tz**, and **BCN-Q** (all at 1 μM) in DMF.

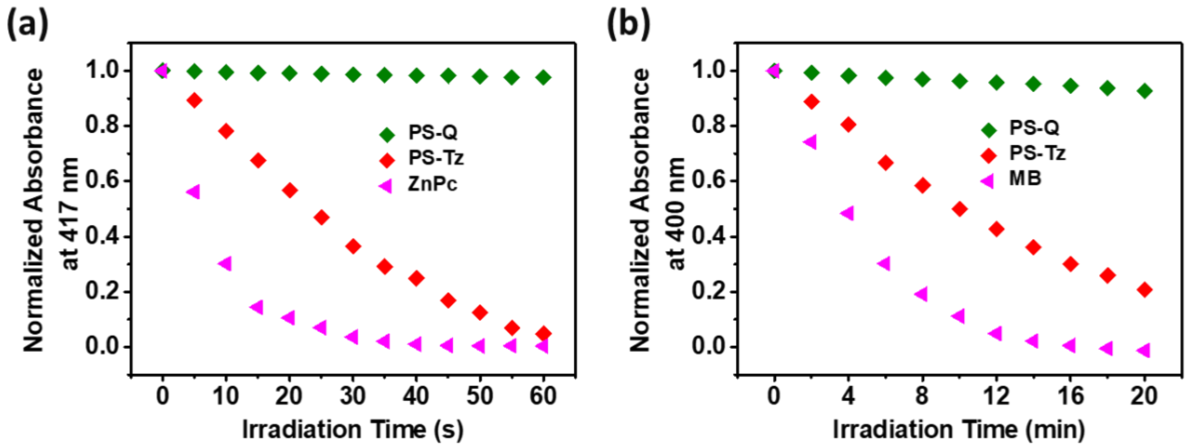

Figure S9. (a) Comparison of the rates of decay of DPBF in DMF (initial concentration = 30 μM), as monitored spectroscopically at 417 nm, using **PS-Q**, **PS-Tz**, and ZnPc (all at 1 μM) as the photosensitizers. (b) Comparison of the rates of decay of ABDA in water with 0.1% Tween 80 (v/v) (initial concentration = 30 μM), as monitored spectroscopically at 400 nm, using **PS-Q**, **PS-Tz**, and MB (all at 1 μM) as the photosensitizers.

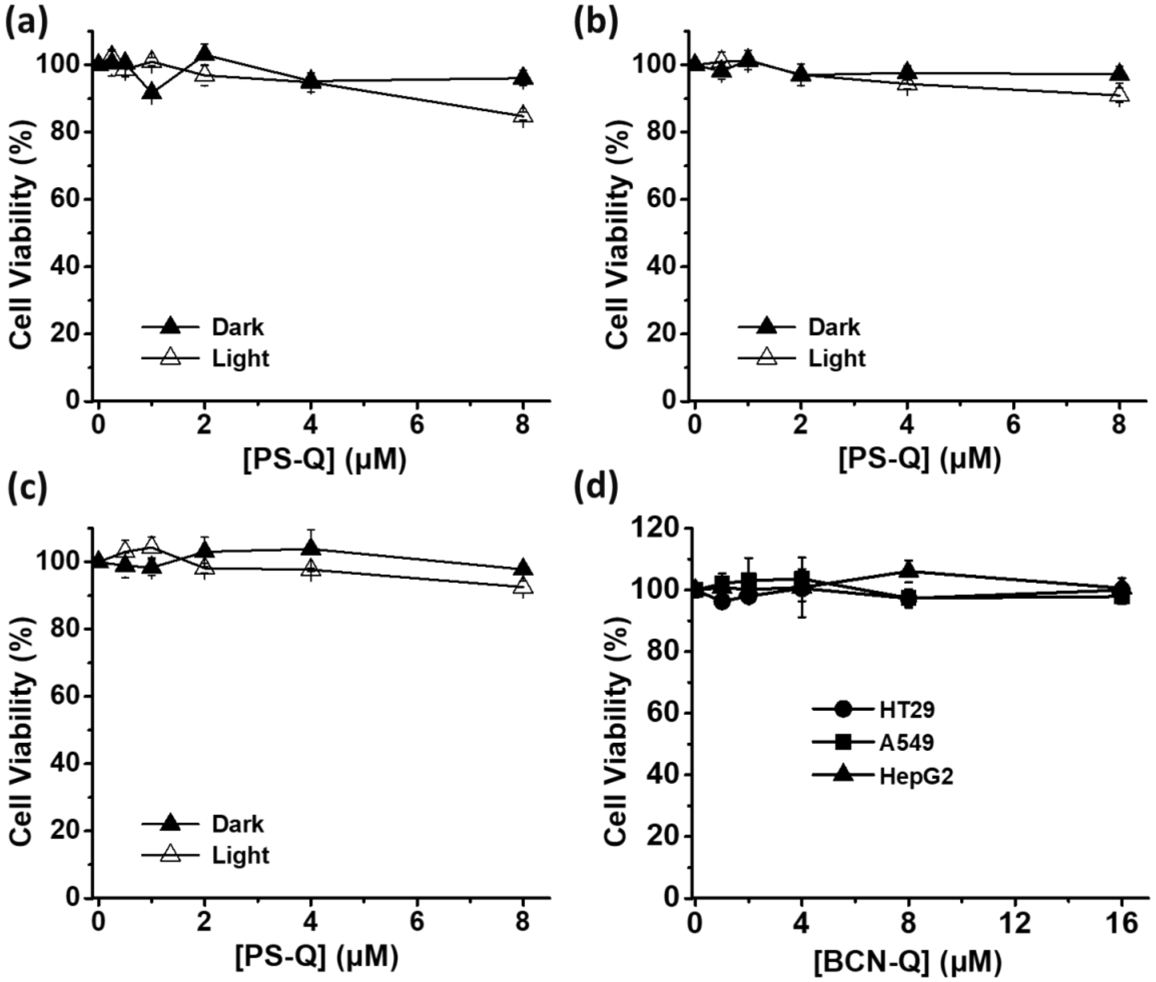

Figure S10. Cytotoxicity of **PS-Q** against (a) HT29, (b) A549, and (c) HepG2 cells in the absence and presence of light irradiation (λ > 610 nm, 23 mW cm^{-2}) for 20 min. (d) Dark cytotoxicity of **BCN-Q** against HT29, A549, and HepG2 cells. Data are reported as the mean ± SEM of three independent experiments, each performed in quadruplicate.

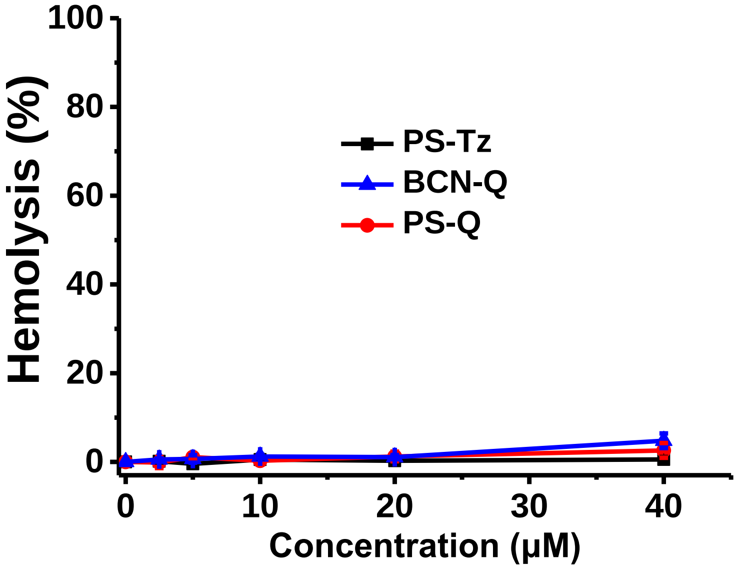

Figure S11. Hemolytic activity of the photosensitizer **PS-Tz**, the antidote **BCN-Q**, and the conjugate **PS-Q** against rabbit red blood cells after incubation at 37 °C for 6 h.

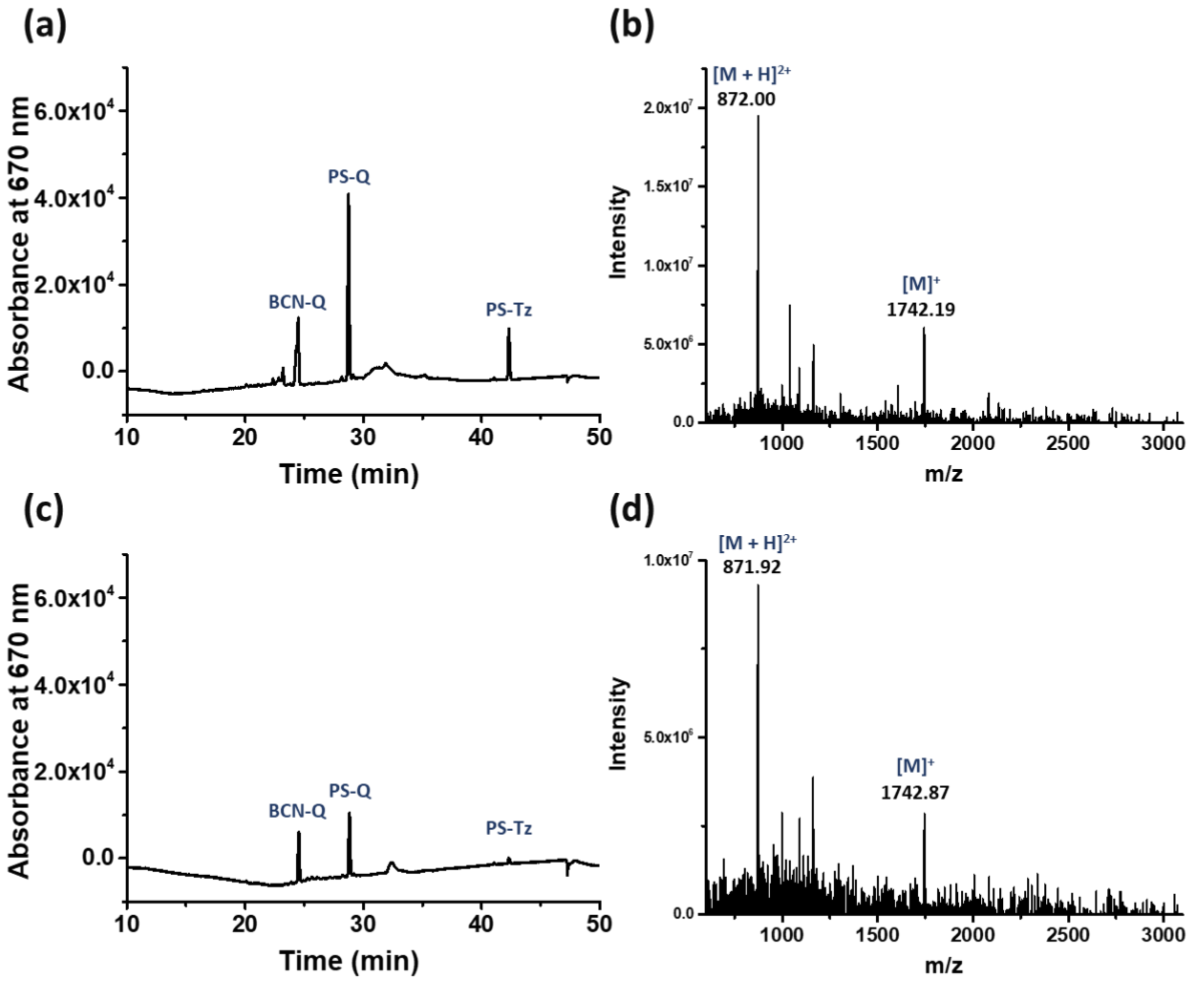

Figure S12. HPLC chromatograms of the blood samples collected at (a) 10 min and (c) 8 h post-injection of **BCN-Q**. The mice were first intravenously injected with **PS-Tz** in PBS in the presence of 0.1% Tween 80 (v/v) (20 nmol, 100 μL), followed by intravenous injection of **BCN-Q** in PBS (40 nmol, 200 μL) at 24 h post-injection of **PS-Tz**. (b,d) ESI mass spectra of the corresponding fractions with a retention time of 28.7 min.

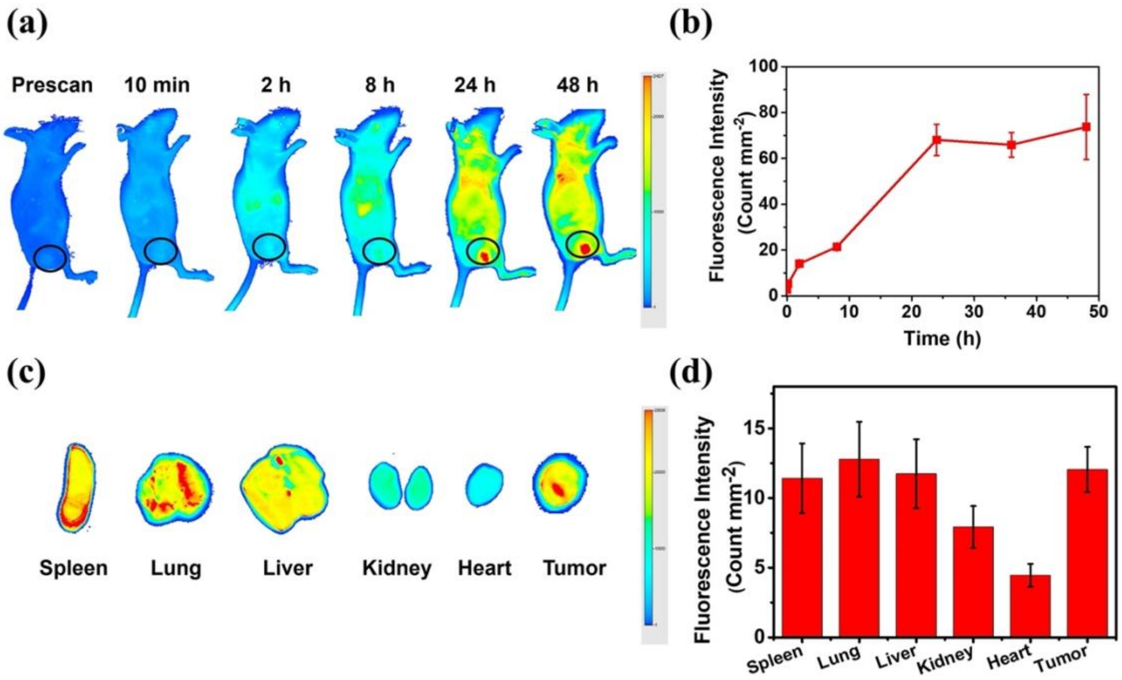

Figure S13. (a) Near-infrared (λ ≥ 700 nm) fluorescence images of HT29 tumor-bearing nude mice after intravenous injection with **PS-Tz** in PBS in the presence of 0.1% Tween 80 (v/v) (20 nmol, 100 μL) over a period of 48 h. (b) Change in fluorescence intensity per unit area of the tumor in the **PS-Tz**-treated mice along with time. (c) Ex vivo images and (d) quantified fluorescence intensities of the tumor and some major organs harvested from the mice at 48 h post-injection of **PS-Tz**. For (b) and (d), data are reported as the mean ± SD of four mice in each group.

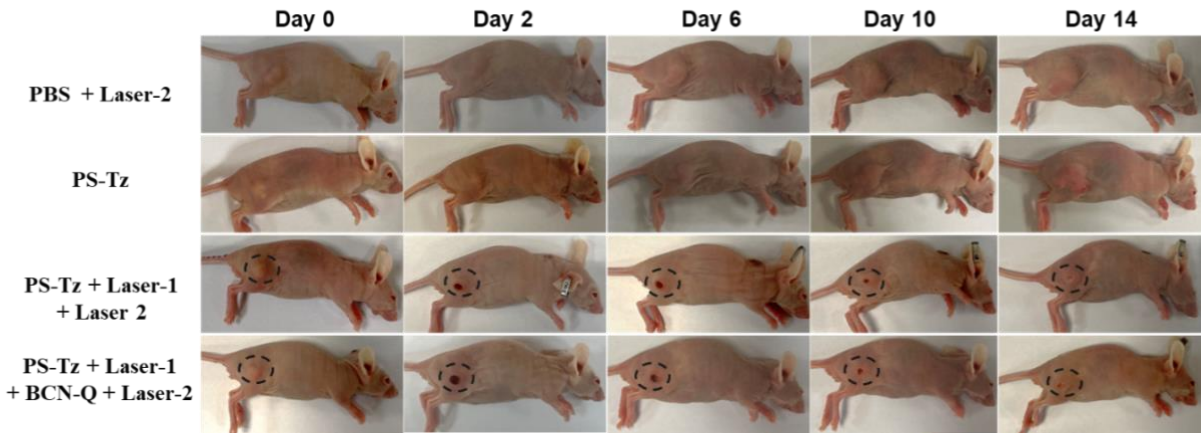

Figure S14. Representative photographs of the mice before and after different treatments: (1) intravenous injection with PBS with the treatment of Laser-2, (2) intravenous injection with **PS-Tz** without laser irradiation, (3) intravenous injection with **PS-Tz** followed by the treatment of Laser-1 and Laser-2, and (4) intravenous injection with **PS-Tz** with the treatment of Laser-1, followed by intravenous injection with **BCN-Q** with the treatment of Laser-2. The irradiated sites of Laser-1 are indicated with ovals. Drug dose: 100 µL of **PS-Tz** (20 nmol) in PBS in the presence of 0.1% Tween 80 (v/v); 200 µL of **BCN-Q** (40 nmol) in PBS. Laser-1: 675 nm, 0.3 W, 180 J cm^{-2}; Laser-2: 675 nm, 0.6 W, 360 J cm^{-2}.

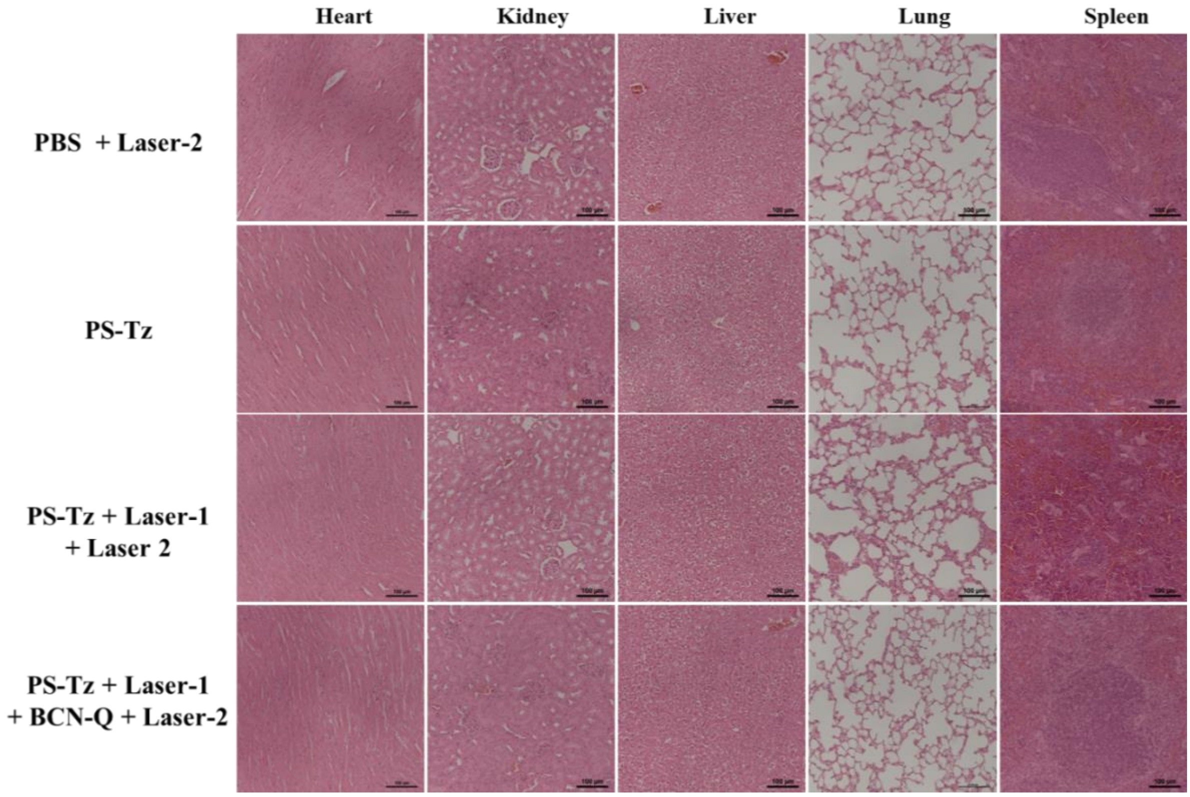

Figure S15. H&E-stained images of different organ slides from the mice scarified on Day 14 after different treatments.

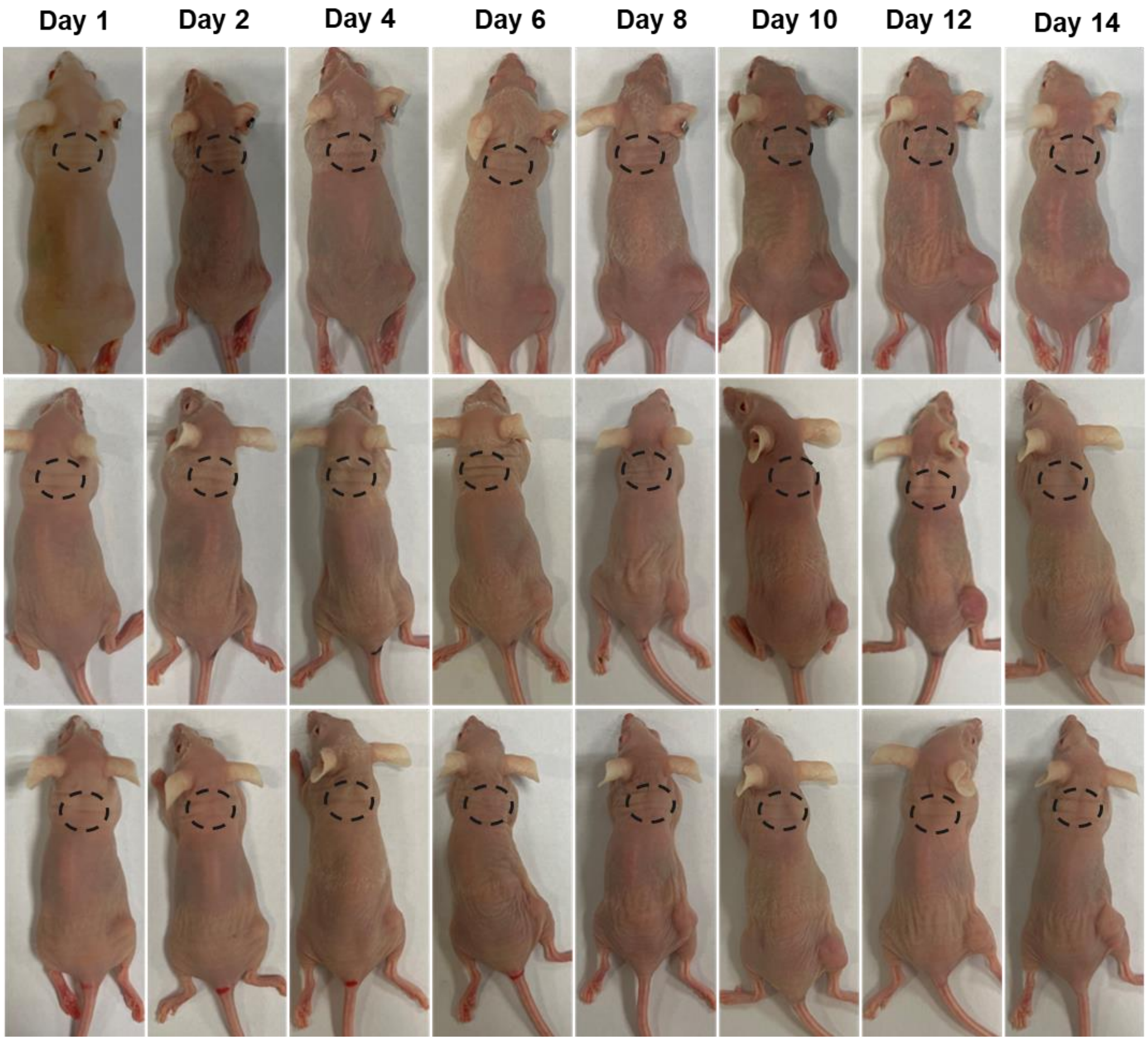

Figure S16. Photographs of the other three mice of group 1, i.e., with intravenous injection with PBS and irradiation with Laser-2 (675 nm, 0.6 W, 360 J cm^{-2}.), over a period of 14 days. The irradiated sites of Laser-2 are indicated with ovals.

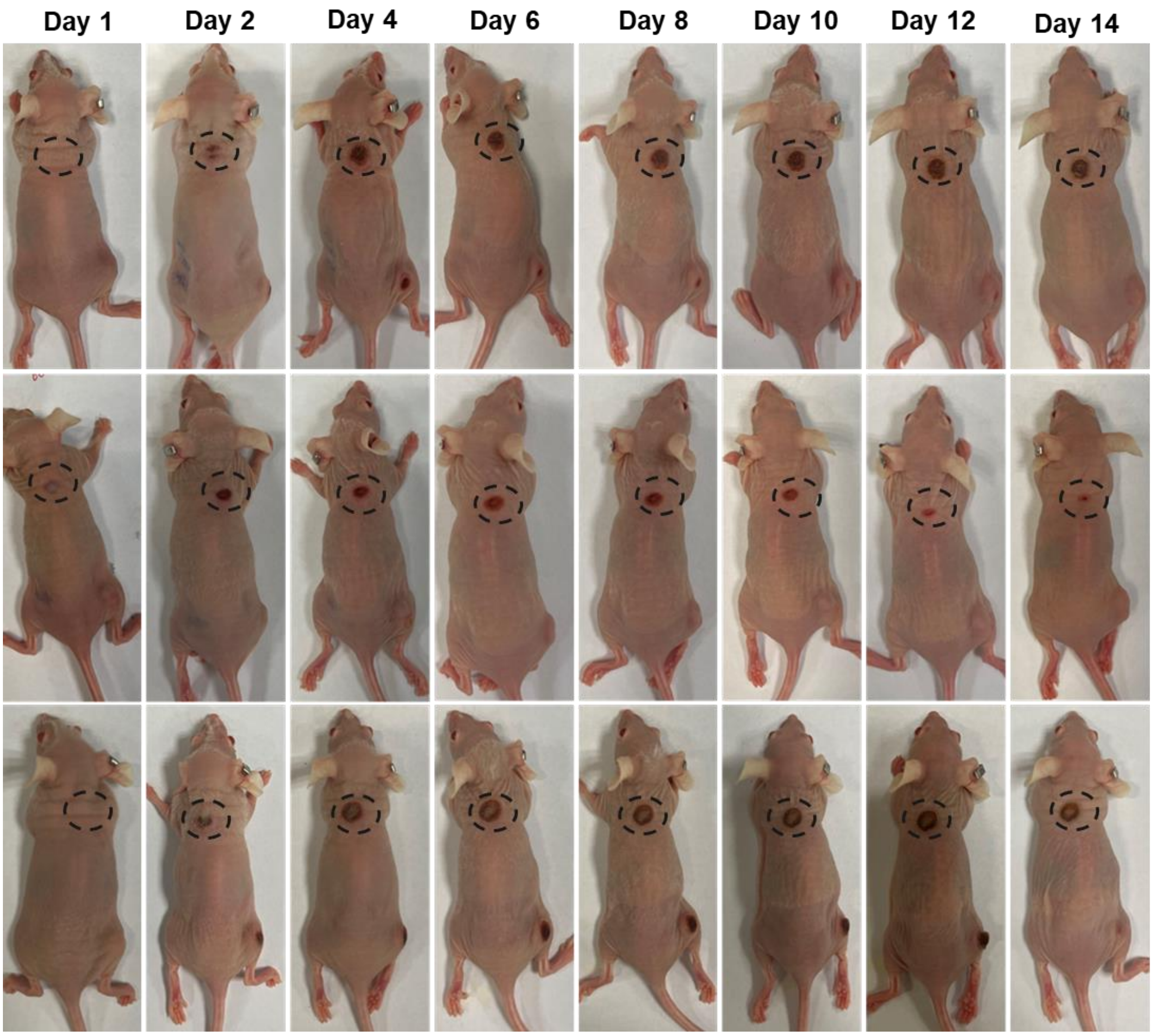

Figure S17. Photographs of the other three mice of group 3, i.e., with intravenous injection with **PS-Tz** (20 nmol) followed by irradiation with Laser-1 (675 nm, 0.3 W, 180 J cm^{-2}) and Laser-2 (675 nm, 0.6 W, 360 J cm^{-2}), over a period of 14 days. The irradiated sites of Laser-2 are indicated with ovals.

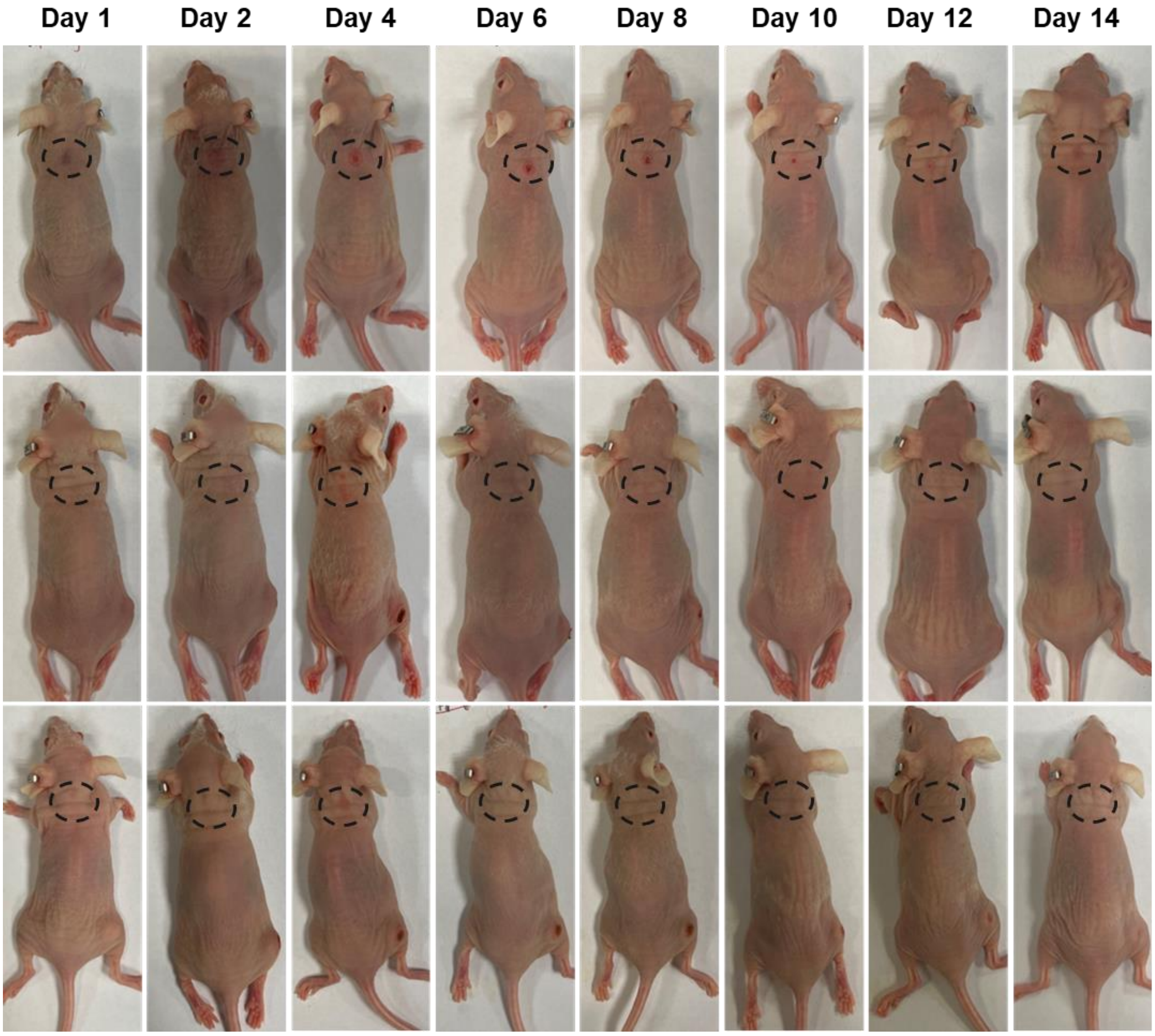

Figure S18. Photographs of the other three mice of Group 4, i.e., with intravenous injection with **PS-Tz** (20 nmol) and irradiation with Laser-1 (675 nm, 0.3 W, 180 J cm^{-2}), followed by intravenous injection with **BCN-Q** (40 nmol) and irradiation with Laser-2 (675 nm, 0.6 W, 360 J cm^{-2}), over a period of 14 days. The irradiated sites of Laser-2 are indicated with ovals.
